# Mamba time series forecasting with uncertainty quantification

**DOI:** 10.1088/2632-2153/adec3b

**Published:** 2025-07-22

**Authors:** Pedro Pessoa, Paul Campitelli, Douglas P Shepherd, S Banu Ozkan, Steve Pressé

**Affiliations:** 1 Center for Biological Physics, Tempe, AZ, United States of America; 2 Department of Physics, Tempe, AZ, United States of America; 3 School of Molecular Sciences—Arizona State University, Tempe, AZ, United States of America

**Keywords:** uncertainty quantification, state space models, non-linear dynamics, Time Series Forecasting

## Abstract

State space models, such as Mamba, have recently garnered attention in time series forecasting (TSF) due to their ability to capture sequence patterns. However, in electricity consumption benchmarks, Mamba forecasts exhibit a mean error of approximately 8%. Similarly, in traffic occupancy benchmarks, the mean error reaches 18%. This discrepancy leaves us to wonder whether the prediction is simply inaccurate or falls within error given spread in historical data. To address this limitation, we propose a method to quantify the predictive uncertainty of Mamba forecasts. To achieve this, we propose a dual-network framework based on the Mamba architecture for probabilistic forecasting, where one network generates point forecasts while the other estimates predictive uncertainty by modeling variance. We abbreviate our tool, Mamba with probabilistic TSF, as Mamba-ProbTSF and the code for its implementation is available on GitHub https://github.com/PessoaP/Mamba-ProbTSF. Evaluating this approach on synthetic and real-world benchmark datasets, we find Kullback–Leibler divergence between the learned distributions and the data–which, in the limit of infinite data, should converge to zero if the model correctly captures the underlying probability distribution–reduced to the order of 10^−3^ for synthetic data and 10^−1^ for real-world benchmark. We find that in both the electricity consumption and traffic occupancy benchmark, the true trajectory stays within the predicted uncertainty interval at the two-sigma level about 95% of the time. We further compare Mamba-ProbTSF against leading probabilistic forecast methods, DeepAR and ARIMA, and show that our method consistently achieves lower forecast errors while offering more reliable uncertainty quantification. We end with a consideration of potential limitations, adjustments to improve performance, and considerations for applying this framework to processes for purely or largely stochastic dynamics where the stochastic changes accumulate as observed, for example, in pure Brownian motion or molecular dynamics trajectories.

## 1. Introduction

Time series forecasting (TSF) is the task of predicting how to complete sequences. In other words, inferring (or forecasting) additional elements of a sequence given a subset of its preceding elements [1–3]. At its core, TSF operates under the assumption that the observed sequence is a realization of some underlying dynamical process [4–6]. This perspective suggests that if we can learn the governing laws driving these dynamics from the available portion of the sequence, the past, we can extend our understanding to forecast the future.

Mathematically, we define the lookback horizon of ‘past’ values as follows: $\{x_1, x_2, \ldots, x_P\}$, and abbreviate to $x_{1:P}$. Our goal is to predict the next *T* (the forecast horizon) values, denoted $x_{P+1:P+T}$. This is achieved through a function represented by a *T*-variate forecast function ${f_{1:T}}(x_{1:P}) = \left(f_1(x_{1:P}), \ldots, f_T(x_{1:P})\right)$, such that $x_{P+\tau} \approx f_\tau(x_{1:P})$. This can be achieved with an explicit dynamical model [7]; for example, in the case of a free-falling particle, we can use past observations to calculate parameters such as initial velocity, gravitational acceleration, and relevant drag coefficients [8]. However, in many fields where TSF is applied—such as finance [1] and climate science [9, 10]—we lack access to explicit dynamical models, or the governing equations may be too complex to describe analytically.

Under these scenarios, rather than assuming a predefined model, we attempt to learn the model. For this task, neural networks (NNs) provide a framework capable of capturing high-dimensional relationships within the data [11]. These NNs are trained by being exposed to multiple realizations of the same underlying dynamical process, observing sequences of past and future data points, and optimizing a convex loss function of the difference between the predicted and the actual future values.

**Figure 1.**
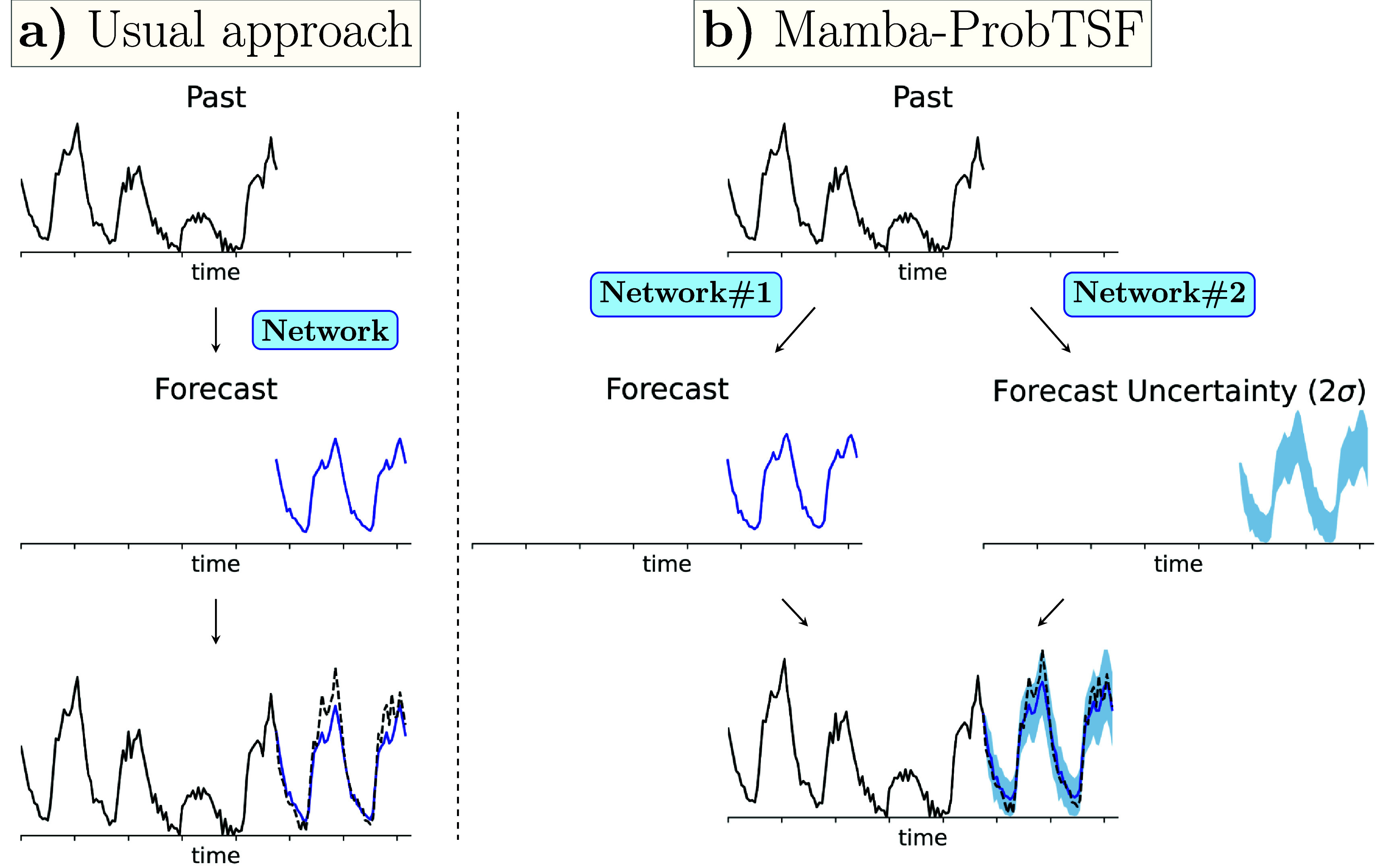
Summary of our dual-network approach in Mamba-ProbTSF. (a) The standard deterministic approach and (b) our proposed dual-network probabilistic approach. In the standard approach, a single neural network forecasts future values directly from historical data, typically minimizing point-wise errors. In our approach, two separate neural networks are employed: one forecasts the future trajectory, while the other estimates the associated uncertainty by leveraging correlations across the dataset at different time points observed during training. As shown in this example—derived from the electricity dataset analysis detailed in section 3.3—this structure enables smoother forecasts by appropriately attributing variability to uncertainty. As a result, the real future trajectory (dashed black line) lies within the uncertainty forecast.

More precisely, the NN approximates the forecast function as ${f_{1:T}}^\phi(x_{1:P})$ where *φ* represents the internal network parameters. These parameters are optimized to minimize a loss function, such as $\sum\nolimits_{\tau = 1}^T \left(x_{P+\tau} - f^\phi_\tau(x_{1:P}) \right)^2. $ This approach is summarized in figure 1(a).

The form of *f*^*φ*^ is determined by the network architecture. While selecting the appropriate architecture remains a challenging task in the field of NNs, state space models (SSMs) – and in particular, the Mamba architecture [12, 13]—have emerged as highly successful for TSF [14–16]. Mamba combines the efficiency of SSMs with an advanced selective mechanism akin to attention [17] outperforming transformer-based architectures in both accuracy and computational efficiency [14].

Though these models provide point predictions for future values, they do not inherently quantify the uncertainty associated with these predictions. Under many practical scenarios, knowing how much one can trust a prediction is just as important as the prediction itself, as shown in examples of biochemical network kinetics [18–20] and more general dynamical systems [4–6]. These arguments, in turn, motivate our probabilistic extension of Mamba.

Existing NN approaches that yield non-deterministic forecasts are often generative models, where repeated evaluations of the same input produce distinct future trajectories [21–23]. While this variability captures predictive uncertainty, it is unclear whether the differences between trajectories represents uncertainty in the underlying dynamics [24]. Thus, there is no guarantee that the spread of trajectories aligns with the uncertainty in the dynamics under study informed from historical data. Therefore, rather than predicting multiple possible trajectory continuations, the objective should be to learn a probability distribution over the ‘future’ conditioned on the observed ‘past’ $p(x_{P+1:P+T}| x_{1:P})$. In other words, develop a probabilistic TSF [24–26].

In this work, we adopt a more structured approach to TSF in a probabilistic manner by introducing a dual-network architecture designed to estimate both the forecasted trajectory and its forecasted uncertainty explicitly thus making it possible to calculate probability densities of the forecast given the past. The implementation, termed Mamba-ProbTSF, is available in our GitHub repository [27]. Specifically, we define two functions

\begin{eqnarray*} {\mu_{1:T}}^{\phi_\mu}\left(x_{1:P}\right) &amp; = \left(\mu^{\phi_\mu}_1\left(x_{1:P}\right), \ldots, \mu^{\phi_\mu}_T\left(x_{1:P}\right)\right), \quad \mathrm{and}\end{eqnarray*}

\begin{eqnarray*} {\sigma_{1:T}}^{\phi_\sigma}\left(x_{1:P}\right) &amp; = \left(\sigma^{\phi_\sigma}_1\left(x_{1:P}\right), \ldots, \sigma^{\phi_\sigma}_T\left(x_{1:P}\right)\right),\end{eqnarray*}

where $\mu^{\phi_\mu}_\tau$ represents the predicted (or forecast) value at step *τ*, and $\sigma^{\phi_\sigma}_\tau$ represents the associated uncertainty (or forecast uncertainty) at step *τ*. To enforce probabilistic consistency, we assume that the conditional distribution of future values is Gaussian centered at the predicted mean with variance given by the squared predicted standard deviation—that is $x_{P+\tau} | x_{1:P} \sim \mathrm{Normal}\left(\mu^{\phi_\mu}_\tau (x_{1:P}), \left(\sigma^{\phi_\sigma}_\tau (x_{1:P}) \right)^2\right)$. A summary of this approach is given in figure 1(b). Any correlation between future points is determined by the past, as the future values $x_{P+\tau}$ are assumed conditionally independent given $x_{1:P}$. In other words, the covariance between different time steps *τ* is assumed to be diagonal.

In the following sections, we evaluate the proposed architecture using both synthetic and real-world datasets. This is done by analyzing the standardized residuals—meaning the difference between the real future data $x_{P:P+T}$, and the forecast ${\mu_{1:T}}^{\phi_\mu}(x_{1:P})$, divided by the forecast uncertainty ${\sigma_{1:T}}^{\phi_\sigma}(x_{1:P})$, later defined in section 2.4—across a testing dataset. Ideally, when the network is properly trained and the stochasticity in the underlying model is indeed Gaussian, as discussed in the examples of section 3, these standardized residuals should follow a standard Gaussian distribution. To quantify the model’s performance, we examine the variance of these standardized residuals and compute their Kullback–Leibler (KL) divergence from a standard normal distribution. In what follows, we observe the success of this approach as well as its limitations.

## 2. Methods

### 2.1. Related work

TSF has been tackled using a variety of models differing in how they represent temporal dependencies and whether they quantify predictive uncertainty.

A foundational method in TSF is the autoregressive integrated moving average (ARIMA) [7, 28] expressing the current value of a series as a linear combination of its recent past values and previous forecast errors. Probabilistic extensions of ARIMA are available in some libraries, where the forecast error distribution is modeled explicitly [29, 30]. However, ARIMA treats each trajectory independently and is inherently limited in capturing nonlinear dynamics due to its linear formulation. When multiple trajectories are assumed to originate from the same underlying process, it becomes advantageous to implement methods able to learn dynamical laws shared by all examples, such as neural networks.

A widely used neural network-based approach for probabilistic TSF is DeepAR [21], which relies on recurrent neural networks (RNNs), particularly the long short-term memory (LSTM) architecture, designed to capture long-term dependencies [31, 32]. While effective, RNNs suffer from sequential processing constraints that limit parallelization and scalability on long sequences.

In parallel, we have seen other advances in non-probabilistic methods. Several alternative neural architectures have been proposed with three notable classes being: SSMs, Kolmogorov–Arnold networks (KANs), and Transformer-based architectures. SSMs model temporal dynamics by learning a latent representation that evolves according to a differential or difference equation [33, 34] (see section 2.2). A key advantage is their ability to operate with linear time complexity in sequence length [34]. KANs implement the Kolmogorov–Arnold representation by learning functional mappings through spline bases for each input dimension [35]. This enhances both expressiveness and interpretability, and recent extensions to sequential forecasting have shown promising results [36]. Transformer-based models use self-attention mechanisms [17] to capture long-range dependencies without recurrence. This allows each time step to directly weigh the relevance of all others, enabling full parallelism. Informer [37], Autoformer [38], and FEDformer [39] adapt this framework to TSF by sparse attention, seasonal trend decomposition, and frequency domain filtering, respectively.

The tradeoffs between these architectures have led to the development of the Mamba architecture [13], which combines SSMs with selective attention mechanisms. Like transformers, they can model long-range temporal dependencies, while maintaining linear time complexity with respect to sequence length, offering improved scalability. A Mamba-based architecture tailored for TSF, S-Mamba [14], was recently benchmarked against transformer-based models and showed superior performance on deterministic forecasting tasks.

Although a full architectural description of S-Mamba is beyond the scope of this work, two of its design elements are particularly relevant for understanding its success: First, each encoder layer includes two Mamba blocks applied in opposite directions (forward and backward in time). Their outputs are added to the input, allowing the model to integrate information from both past and future. As known from Markovian inference [4, 6], access to full trajectories can improve parameter estimation. Ablation studies in [14] confirm that this bidirectional design consistently outperforms unidirectional variants.

Second, S-Mamba uses a transposed embedding strategy: the input is reshaped so that each time series is treated as an independent sequence, while sharing the same dynamical structure. This enables the Mamba operator to model temporal dependencies directly within each trajectory, avoiding interference between time and feature dimensions. The result is improved learning of trajectory specific patterns.

In this work, we extend S-Mamba by introducing a second network that predicts the uncertainty in future values. This results in Mamba-ProbTSF, a model that retains the computational advantages of S-Mamba while enabling direct and interpretable uncertainty quantification. Table 1 summarizes key architectural differences and indicates whether each method supports predictive uncertainty or attention mechanisms. This discussion also motivates the next section, where we describe the state-space models, along with the structural assumptions that guide our probabilistic formulation.

**Table 1.** Comparison of forecasting models by architecture type, use of attention, and support for predictive uncertainty. Details in section 2.1.

Method	Architecture type	Attention	Uncertainty quantification
ARIMA [7]	Linear statistical	No	Limited
DeepAR [21]	RNN	No	Yes
Informer [37]	Transformer variant	Yes	No
Autoformer [38]	Transformer variant	Yes	No
FEDformer [39]	Transformer variant	Yes	No
KAN [36]	Functional basis	No	No
S-Mamba [14]	SSM	Yes	No
Mamba-ProbTSF (this paper)	SSM	Yes	Yes (mean + std)

### 2.2. State-space models

Here we recall the basics of SSMs. While a comprehensive review of all SSM architectures is beyond the scope of this article, we build upon the overarching summary provided in the previous paragraph to clarify the mathematical foundations and implicit assumptions underlying Mamba. This discussion helps contextualize Mamba’s applicability and supports the interpretation of results presented in section 3.

SSMs operate under the premise that the observed data is influenced by an underlying latent process, which the model attempts to infer. To describe SSMs in a general context, we index the input, $\mathcal{I}_t^n$, with *t* denoting time and *n* denoting different trajectories in the dataset each corresponding to a distinct realization of a same underlying dynamical process.

The SSM produces the output $\mathcal{O}_t^n$ in two steps. The first step applies a differential equation model to the latent variable *h^n^*:

\begin{equation*} \frac{\mathrm{d}h^n\left(t\right)}{\mathrm{d}t} = \sum_{m = 1}^N \left( A^n_m h^m\left(t\right) + B^n_m \mathcal{I}_t^m \right),\end{equation*}

where *N* denotes the number of trajectories in the input and both $A^n_m$ and $B^n_m$ are learned matrices, meaning each element is a parameter of the neural network optimized during training. The sum over *m* accounts for interactions across multiple latent variables, reflecting a coupled system where each trajectory *n* is influenced not only by its own past but also by shared information from others. This allows the model to leverage dependencies across different trajectories particularly useful for time series exhibiting common underlying dynamics. The second step projects the latent state onto the output space

\begin{equation*} \mathcal{O}_t^n = \sum_{m = 1}^N C^n_m h^m\left(t\right),\end{equation*}

where $C^n_m$ is another learnable matrix responsible for the final projection.

In practice, these continuous-time equations must be discretized for implementation in modern neural network architectures. The discretization step transforms (4) into:

\begin{equation*} h^n_{t+1} = \sum_{m = 1}^N \left( \tilde{A}^n_m h^m_t + \tilde{B}^n_m \mathcal{I}_t^m\right),\end{equation*}

where $\tilde{A}^n_m$ and $\tilde{B}^n_m$ are discrete approximations of the continuous parameters, given in terms of $A^n_m$ and $B^n_m$ through the matrix exponential

\begin{eqnarray*} \tilde{A}^n_m = \left[\exp\left(\Delta_t\mathbf{A} \right)\right]^n_m, \quad \mathrm{and} \quad\end{eqnarray*}

\begin{eqnarray*} \tilde{B}^n_m = {\sum_{o = 1}^{N}} \left[ \left(\Delta_t \mathbf{A}\right)^{-1} \left(\exp\left(\Delta_t \mathbf{A}\right) - \mathbf{I}\right) \right]^n_o B^o_m.\end{eqnarray*}

Here, **A** represents the matrix of elements $A^n_m$, **I** is the identity matrix, and $\Delta_t$ is a learnable real-valued discretization step. This transformation ensures numerical stability and preserves the underlying system dynamics over discrete time intervals without assuming that the time between observations is small enough to warrant a first order approximation.

By discretizing the state-space formulation, SSMs can be efficiently implemented in neural architectures, leveraging parallelization techniques to process long sequences while maintaining the model’s structural advantages. Integrating learned parameterizations with discretized updates allows models like Mamba to achieve both flexibility and scalability, making them particularly effective for time-series forecasting tasks that require capturing long-range dependencies. For further details, we refer to the original Mamba authors [12, 13].

### 2.3. Network training

Here, we describe how to train the two networks, representing ${\mu_{1:T}}^{\phi_\mu}(x_{1:P})$ and ${\sigma_{1:T}}^{\phi_\sigma}(x_{1:P})$ in (1), for probabilistic TSF. For the standard architecture for ${\mu_{1:T}}^{\phi_\mu}(x_{1:P})$, we use S-Mamba [14], while for ${\sigma_{1:T}}^{\phi_\sigma}(x_{1:P})$, we use a fully connected neural network with a softplus activation in the last layer to ensure a strictly positive output. The full implementation is available in our GitHub repository [27].

#### 2.3.1. Loss function for probabilistic TSF

Here we discuss the loss function used in Mamba-ProbTSF. As mentioned in the introduction, we assume a Gaussian conditional probability for a point in the ‘future’ conditioned on the ‘past’ given by

\begin{equation*} p\left(x_{P+\tau}|x_{1:P}\right) = \frac{1}{\sqrt{2\pi} \ \sigma^{\phi_\sigma}_\tau\left(x_{1:P}\right)} \exp\left[ - \frac{1}{2} \left( \frac{x_{P+\tau} - \mu^{\phi_\mu}_\tau\left(x_{1:P}\right)}{\sigma^{\phi_\sigma}_\tau\left(x_{1:P}\right)} \right)^2 \right] ,\end{equation*}

and the neural network training consists of finding the parameter sets *φ*_*µ*_ and *φ*_*σ*_ such that ${\mu_{1:T}}^{\phi_\mu}$ and ${\sigma_{1:T}}^{\phi_\sigma}$ that best represent the underlying dynamics.

While (8) assumes conditional independence across forecasted time steps, this does not preclude the model from learning temporal dependencies. Since each forecast $\mu^{\phi_\mu}_\tau(x_{1:P})$ and associated uncertainty $\sigma^{\phi_\sigma}_\tau(x_{1:P})$ are conditioned on the full input history $x_{1:P}$, the outputs for different *τ* can still exhibit structured dependencies. In this sense, temporal correlations within the forecast are not imposed explicitly but can emerge implicitly through the shared conditioning on the past.

Moreover, although the assumption of a Gaussian probability distribution at every point aligns well with many scientifically relevant scenarios – e.g. Brownian motion (further discussed in section 3.6) [40] or deterministic dynamics with Gaussian observational noise (further discussed in sections 3.1 and 3.2) [41, 42]—Maximum entropy also provides a justification for selecting the Gaussian form: the Gaussian distribution is the least biased choice when only the first and second moments of the distribution are specified [43–45]. Thus, even in cases where the Gaussian assumption may not strictly hold, the Gaussian-based loss function (8) allows the dual-network to still learn the predictions mean (forecast) and variance (forecast uncertainty).

We now turn this probabilistic formulation into a concrete loss function for training. We partition some dynamical realizations into a training dataset, where each trajectory is indexed by a superscript $n \in \{1, \ldots , N\}$, such that each trajectory in the dataset is given as $x^n_{1:P+T}$. To estimate the optimal parameters, we maximize the total conditional likelihood, assuming independence between trajectories

\begin{equation*} p\left(x_{P+1:P+T}^{1:N}|x_{1:P}^{1:N}\right) = \prod_{n = 1}^N p\left(x_{P+1:P+T}^n|x_{1:P}^n\right) = \prod_{n = 1}^N \prod_{\tau = 1}^T p\left(x_{P+\tau}^n|x_{1:P}^n\right) .\end{equation*}

This is equivalent to minimizing the negative log-likelihood, leading to the following loss function:

\begin{equation*} \mathcal{L}\left(\phi_\mu,\phi_\sigma\right) = \frac{1}{N} \sum_{n = 1}^N \ \sum_{\tau = 1}^{T} \left[ \frac{1}{2} \left( \frac{x_{P+\tau}^n - \mu^{\phi_\mu}_\tau\left(x_{1:P}^n\right)}{\sigma^{\phi_\sigma}_\tau\left(x_{1:P}^n\right)} \right)^2 + \log \sigma^{\phi_\sigma}_\tau\left(x_{1:P}^n\right)\right] ,\end{equation*}

which applies the full probabilistic forecasting.

#### 2.3.2. Steps of training

With the joint loss function (10) specified, a few more details of the training process are in order. First, when we initialize the networks, we train the network for $\mu^{\phi_\mu}_{1:T}(x_{1:P})$ separately from $\sigma^{\phi_\sigma}_{1:T}(x_{1:P})$.

We pre-initialize the first network parameters, *φ*_*µ*_, by training them as if we had a non-probabilistic TSF procedure. That is, we train the first network by using only a point-wise error as the loss function, meaning we minimize

\begin{equation*} \mathcal{L}_\mathrm{pre}\left(\phi_\mu\right) = \frac{1}{N} \sum_{n = 1}^N \sum_{\tau = 1}^{T} \left( x_{P+\tau}^n - \mu^{\phi_\mu}_\tau\left(x_{1:P}^n\right) \right)^2.\end{equation*}

This initialization ensures that the mean predictor ${\mu_{1:T}}^{\phi_\mu}$ learns a stable estimate of the expected future trajectory before incorporating uncertainty. This is then equivalent to (10) if one assumed that the uncertainty at all times is equal, that is $\sigma^{\phi_\sigma}_\tau(x_{1:P}^n) = C$, for all *τ* and *n*.

The constant uncertainty assumption, however, is only used during the pre-initialization to guide *φ*_*µ*_ toward a reasonable solution. After this step, we move into the full probabilistic training phase, where the internal parameters for both mean and uncertainty networks (*φ*_*µ*_ and *φ*_*σ*_) are jointly optimized using the complete loss (10). Thus, the network ${\mu_{1:T}}^{\phi_\mu}$ is not constrained by the initial assumption of constant uncertainty. Despite this, other strategies for joint training, such as curriculum learning [46, 47] or co-training [35, 48], could be explored in future work to further improve training stability and representation quality.

Once the initialization of *φ*_*µ*_ is complete, we proceed with the full probabilistic training. Since training with all trajectories simultaneously may exceed memory limitations, we employ minibatching. That is, in each training step, we randomly select a batch of *B* trajectories from the dataset and compute the loss:

\begin{equation*} \mathcal{L}_B\left(\phi_\mu,\phi_\sigma\right) = \frac{1}{B} \sum_{n \in \mathcal{B}} \sum_{\tau = 1}^{T} \left[ \frac{1}{2} \left( \frac{x_{P+\tau}^n - \mu^{\phi_\mu}_\tau\left(x_{1:P}^n\right)}{\sigma^{\phi_\sigma}_\tau\left(x_{1:P}^n\right)} \right)^2 + \log \sigma^{\phi_\sigma}_\tau\left(x_{1:P}^n\right) \right]. \quad\end{equation*}

where $\mathcal{B}$ represents the minibatch of size *B*. After computing the minibatch loss, we update parameters on both *φ*_*µ*_ and *φ*_*σ*_ using gradient-based optimization tools within PyTorch.

### 2.4. Metrics of success

A common challenge in probabilistic TSF is to verify whether the learned distribution accurately represents the intrinsic stochastic dynamics under study. However, our data is provided in such a way that, for each given ‘past’ we observe only a single corresponding ‘future.’ In other words, we have only one sample from each conditional probability distribution. As such, some approaches attempt to train networks using synthetic datasets, where multiple future samples are generated for a single past [24]. However, this strategy is not consistent with how data is typically presented in real TSF tasks, where only one realization of the stochastic dynamics is available for each historical sequence.

Here, we adopt a different method to quantify whether the neural network has learned the correct distribution. Fortunately, since our training is based on a Gaussian conditional probability (8), we can define the standardized residual at step *τ*:

\begin{equation*} z_\tau^n = \frac{x_{P+\tau}^n - \mu^{\phi_\mu}_\tau\left(x_{1:P}^n\right)}{\sigma^{\phi_\sigma}_\tau\left(x_{1:P}^n\right)}.\end{equation*}

This quantity represents the normalized deviation of the observed value from the predicted mean, scaled by the predicted standard deviation at step *τ*.

If the Gaussian assumption holds for the dynamics under study and the dual network accurately learn the dynamics, the standardized residuals $z_\tau^n$ should follow a standard normal distribution, with mean 0 and variance 1, for all *τ*. This property provides a straightforward way to assess the model’s performance: we check whether the empirical distribution of $z_\tau^n$ across all *τ* matches the expected standard normal distribution. Here, *n* indexes the trajectories in the testing set, as this evaluation is performed on the test data, not during training. Unlike the previous subsection, where *n* referred to the training data, here we use *n* to denote unseen test samples.

From the standardized residuals $z_\tau^n$, we define two key metrics of success. The first is the variance of $z_\tau^n$, which should ideally approach 1 for each *τ*. The second is the Kullback–Leibler (KL) divergence [11] between the empirical distribution of $z_\tau^n$ and the standard normal distribution, which should ideally approach zero. These metrics provide both a quantitative and qualitative assessment of model performance.

In the following section, when describing each dataset under study, we will present histograms of $z_\tau^n$ at different values of *τ*: specifically, at *τ* = 1, at the maximum $\tau = T$, and at the midpoint $\tau = \lfloor T/2 \rfloor$. Qualitatively, these histograms should resemble the shape of a standard normal distribution. Additionally, we will show how both the variance and KL divergence evolve as a function of *τ*, providing insights into how well the model maintains its probabilistic consistency across different forecast horizons.

## 3. Results

In this section, we discuss the results of the probabilistic TSF approach proposed in the previous section, applied to both real and synthetic datasets. Each dataset will be described in its respective subsection.

### 3.1. Sines (synthetic)

As a first example, we generate a simple synthetic dataset where each data point is constructed as a sum of sine waves with different frequencies, plus independent Gaussian noise. Each trajectory in the dataset is given as

\begin{equation*} x_t^n = 4 \sin\left(\omega_1 t+ \phi^n\right) + \sin\left(\omega_2^n t \right) + \xi^n_t.\end{equation*}

We set $\omega_1 = 2\pi/24$, while for each trajectory, *n*, we have a single $\omega_2^n$ is sampled from an exponential distribution with a mean $ 2\pi/12$ and initial phase *φ*^*n*^ sampled uniformly between 0 and 2*π*. The noise term $\xi^n_t$ is independently drawn from a standard normal distribution for all *t*. To clarify notation, *t* indexes all data points across both the ‘past’ and ‘future’ $(1:P+T)$, whereas in the previous section, *τ* was used to refer specifically to indices within the ‘future’ range, with *τ* running from 0 to *T*.

**Figure 2.**
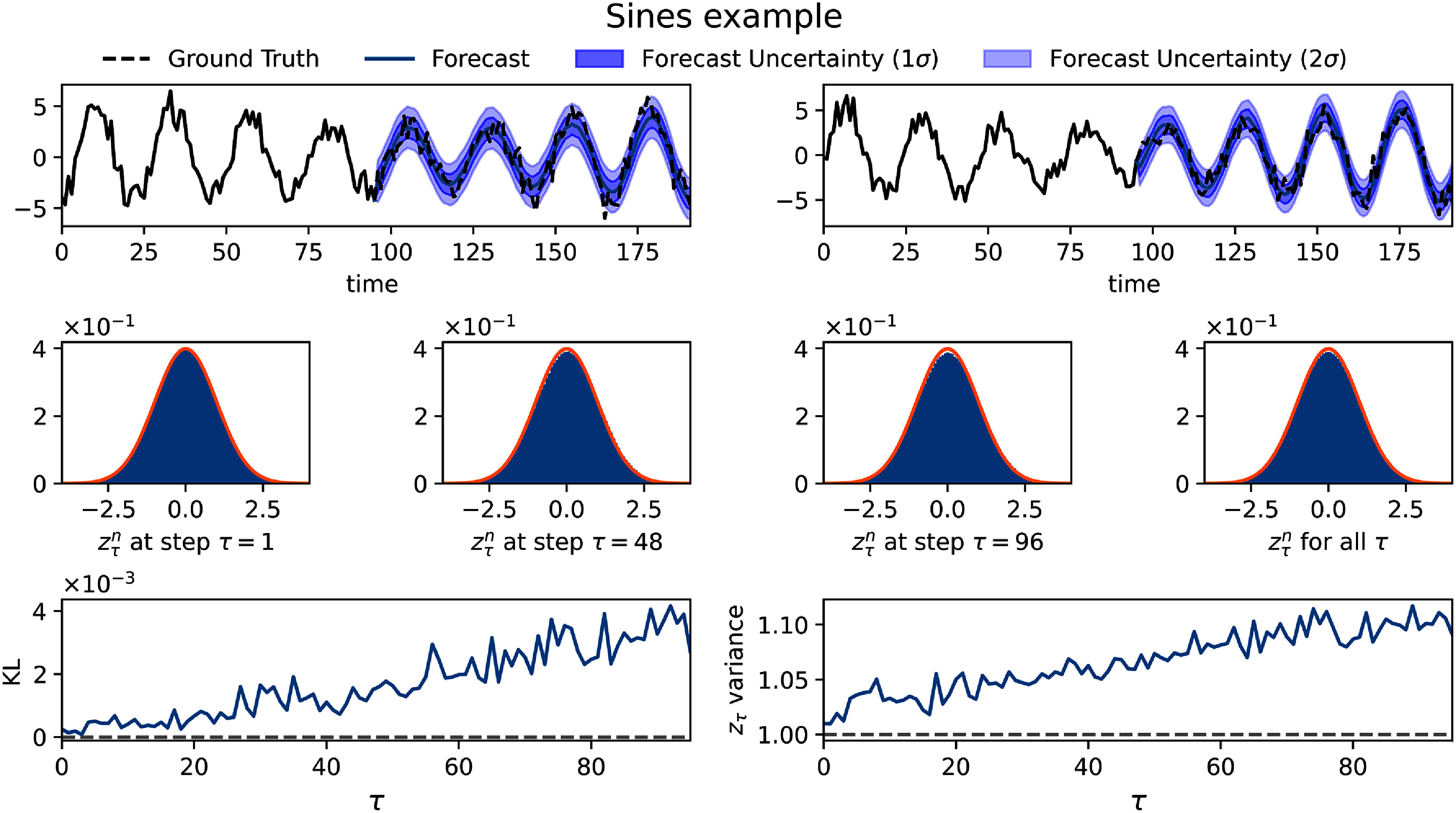
Application of Mamba-ProbTSF to a sum of sines with added Gaussian noise. The first row shows a sample trajectory along with its forecast and predicted uncertainty. The middle row presents histograms of standardized residuals $z_\tau^n$ at the beginning of the forecast (*τ* = 1), the midpoint, the final forecast point (*τ* = 96), and the histogram where we pool all values of $z_\tau^n$ across all *τ*, compared to a standard normal distribution (orange line). The bottom row demonstrates that the variance of $z_\tau^n$ remains close to 1 across different forecast horizons *τ*. The KL divergence between the empirical and standard normal distributions remains low, on the order of 10^−3^.

The results obtained from this dataset are shown in figure 2. We observe that the dual-network system successfully captures both underlying dynamics, as illustrated by the histograms of $z^n_\tau$ matching standard Gaussians, KL approaching 0 (with values of the order of 10^−3^) and variance near 1.

### 3.2. Van der Pol dynamics (synthetic)

As a second synthetic example, we generate a dataset based on the van der Pol oscillator [49], which exhibits nonlinear deterministic dynamics and is a prototype model for systems with self-excited limit cycle oscillations and has been applied to various physical and biological phenomena [50–52].

Each trajectory in the dataset is obtained by numerically integrating the van der Pol differential equation for an underlying variable *y*(*t*)

\begin{equation*} \frac{\mathrm{d}^2 y}{\mathrm{d}t^2} - \omega_1^2 \left(\lambda^n \left(1 - y^2\right) \frac{\mathrm{d}y}{\mathrm{d}t} + y \right) = 0.\end{equation*}

We set $\omega_1 = 2\pi/24$, while for each trajectory *n*, the damping coefficient *λ*^*n*^ is sampled from an exponential distribution with mean 5. The system is numerically integrated using a fixed time step, with initial conditions *y*_0_ = 0 and $\mathrm{d}y/\mathrm{d}t \vert_{t = 0} = 1$. The observed dataset is then constructed by sampling the integrated solution at integer time steps and adding independent Gaussian noise

\begin{equation*} x_t^n = 4 y_t^n + \xi_t^n,\end{equation*}

where $\xi_t^n$ is sampled independently from a standard normal distribution for all *t*.

This setup mirrors the approach used in the sines example, where the observed data combines a deterministic underlying process with stochastic noise. Unlike the sines example, which follows simple harmonic motion, the van der Pol system exhibits nonlinear oscillations where the amplitude and frequency depend on the damping parameter *λ*^*n*^. This makes it a valuable benchmark in testing forecasting models on more complex, state-dependent dynamics.

**Figure 3.**
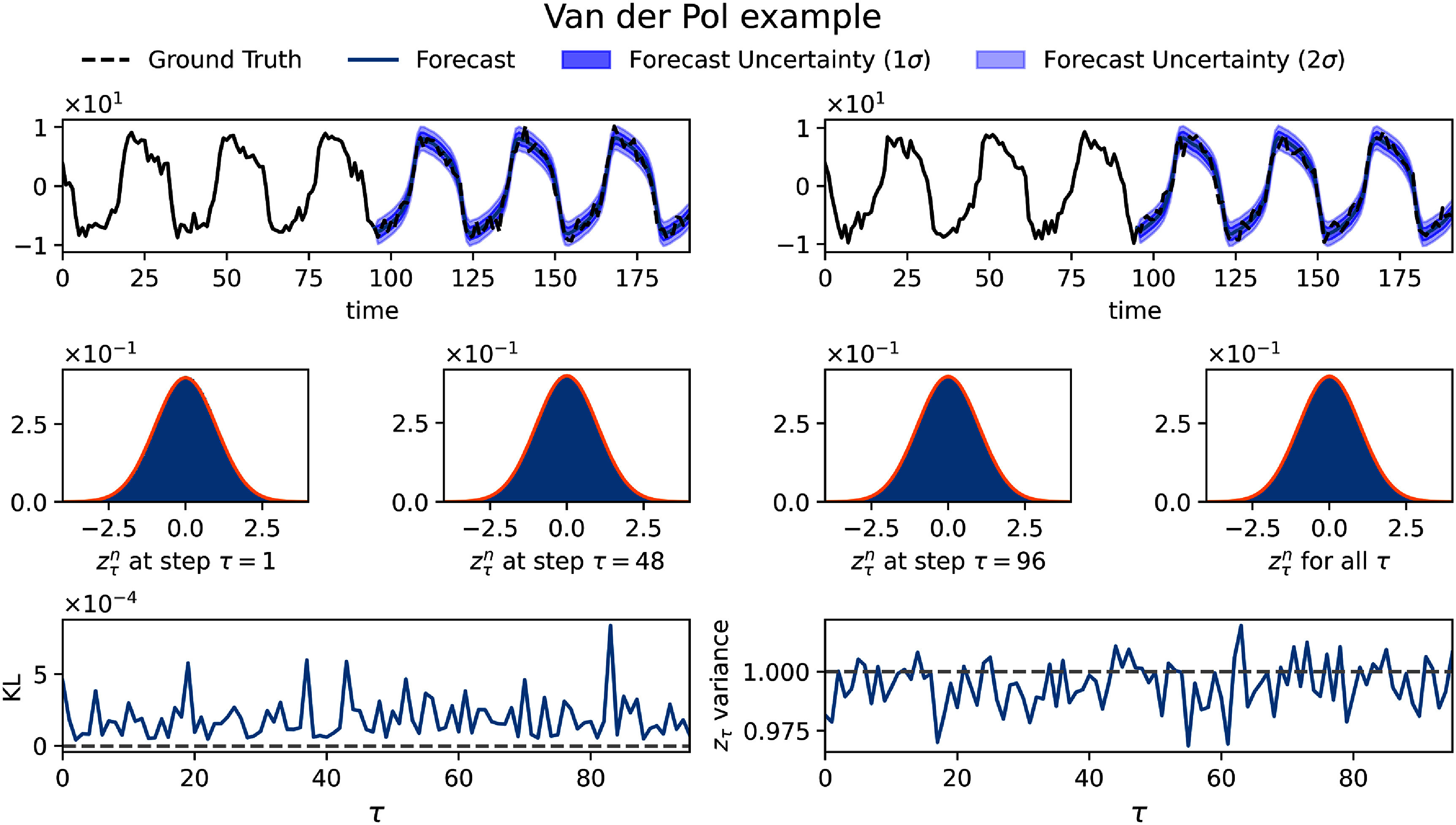
Application of Mamba-ProbTSF to the van der Pol oscillator with added Gaussian noise. Similarly to figure 2, the first row shows a sample trajectory with its forecast and predicted uncertainty. The middle row presents histograms of standardized residuals $z_\tau^n$ at *τ* = 1, the midpoint, *τ* = 96, and all values of *τ* pooled together compared to a standard normal distribution (orange line). The bottom row shows that the variance of $z_\tau^n$ remains close to 1 across different forecast horizons *τ*. The KL divergence between the empirical and standard normal distributions is even lower than in the sines example, on the order of 10^−4^.

The results obtained from this dataset are shown in figure 3. Compared to the sines example, we observe that the dual-network system successfully captures the more complex underlying dynamics. This is reflected in the histograms of $z^n_\tau$, where the KL divergence approaches 0 (on the order of 10^−4^), and the variance remains close to 1, similar to the sines example.

### 3.3. Electricity consumption (real-world)

As a real-world example, we consider the electricity dataset [53], a widely used benchmark in TSF [14, 38, 54]. This dataset records the hourly electricity consumption of 321 customers from 2012 to 2014, originally collected at fifteen-minute intervals [54] and later aggregated for consistency in analysis.

Unlike synthetic datasets, which follow predefined equations, electricity consumption arises from a complex and partially observable dynamical system. The underlying dynamics are influenced by multiple interacting factors, including consumer behavior, economic activity, weather conditions, and energy policies, all of which evolve over time. Despite this complexity, the system exhibits periodicity at daily and weekly scales due to human routines, alongside irregular fluctuations driven by external influences.

**Figure 4.**
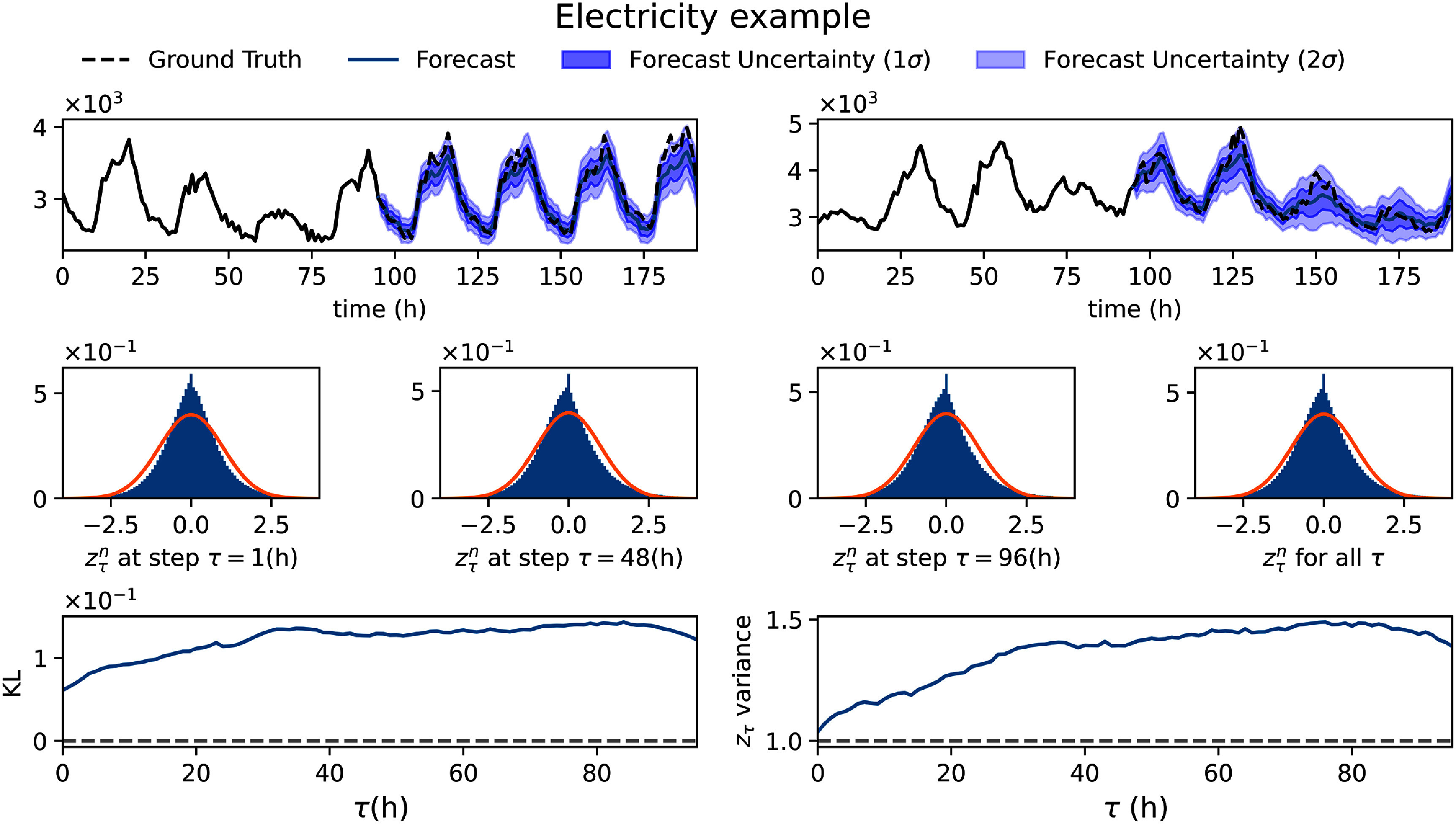
Mamba-ProbTSF applied to the electricity real-world dataset highlights the need for uncertainty modeling. The figure presents results using a fully connected neural network for $\sigma^{\phi_\sigma}_\tau(x_{1:P})$, ensuring stable uncertainty estimates. The probabilistic TSF approach achieves a close match between the standardized residuals, $z_\tau^n$, and the expected standard normal distribution, with a KL divergence on the order of 10^−1^. The Mamba-ProbTSF does not attempt to explicitly separate epistemic uncertainty from variability due to unobserved external factors; instead, it captures overall predictive uncertainty as learned from the training dataset. Despite this, the variance remains within a reasonable range, varying with *τ* but staying below 1.5, demonstrating a well-learned uncertainty function.

In figure 4, we present the results of the dual-network strategy for the electricity dataset. We observe that the KL divergence between standardized residuals $z_\tau^n$ and a standard normal distribution remains stable, on the order of 10^−1^, indicating that the learned uncertainty quantification aligns well with the empirical data. Additionally, the variance of $z_\tau^n$ across different forecast horizons remains below 1.5, demonstrating a consistent and reasonable estimate of forecast uncertainty.

**Figure 5.**
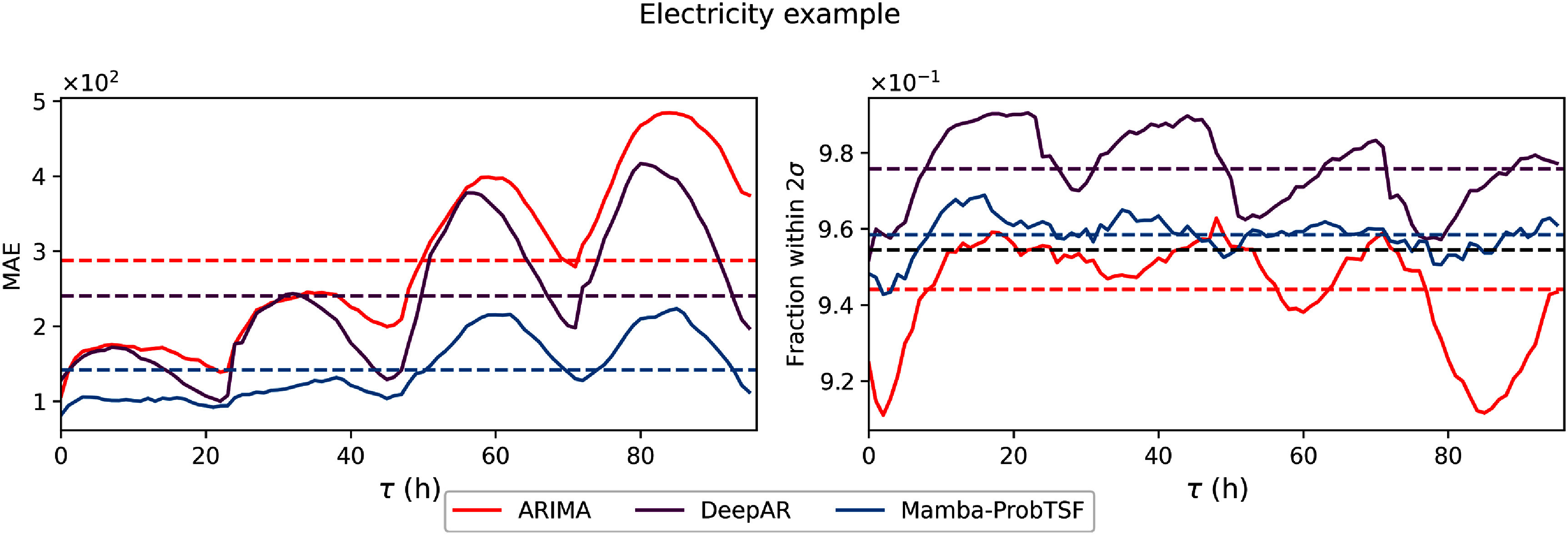
Probabilistic forecasting provides strong error quantification. (Left) Mean absolute error (MAE) on the Electricity dataset. Mamba-ProbTSF consistently outperforms both ARIMA and DeepAR, achieving lower errors across the entire forecast horizon. (Right) Fraction of trajectories falling within the 2*σ* prediction interval for each method. Solid lines show the empirical fraction at each forecast step, while dashed lines of the same colors indicate the time-averaged fraction for each method. The dashed black line marks the expected 95.4% fraction under the Gaussian assumption. Although all methods tend to overestimate the uncertainty range, Mamba-ProbTSF yields a fraction much closer to the expected value and does so more consistently across time. ARIMA shows a similar time-averaged value but with large fluctuations, where Mamba-ProbTSF maintains more stable and interpretable uncertainty estimates throughout the forecast window.

To evaluate the reliability of uncertainty estimates, we measure the fraction of trajectories in the test dataset that fall within the forecast uncertainty bounds (figure 5). Under the assumption that the underlying distribution is Gaussian and the network has accurately learn the correct functions for both the forecast and its uncertainty functions, we would expect approximately 68.3% of data points to lie within the 1*σ* interval ($\mu^{\phi_\mu}_\tau(x_{1:P}^n) \pm \sigma^{\phi_\sigma}_\tau(x_{1:P}^n)$), and 95.4% within the 2*σ* interval.

When benchmarking Mamba-ProbTSF on the Electricity dataset, we find that actual trajectories fall within the 1*σ* interval 78.7% of the time, and within the 2*σ* interval 95.6% of the time, on average. This suggests that when applied to real-world data, where the Gaussian assumption in (8) is not strictly valid, the Gaussian-based loss function tends to provide a conservative estimate of uncertainty.

It is important to compare the accuracy of both the point forecasts and the uncertainty quantification of Mamba-ProbTSF against other probabilistic TSF methods, particularly ARIMA and DeepAR. In figure 5, we present the mean absolute error (MAE) of the point forecasts from ARIMA, DeepAR, and Mamba-ProbTSF, along with the fraction of trajectories that fall within the 2*σ* prediction interval. Further details on the comparison procedure are provided in section 3.5. We observe that Mamba-ProbTSF not only achieves a lower MAE but also produces uncertainty bounds with a fraction of trajectories within the 2*σ* interval (95.8%) that is closest to the expected 95.4%. In comparison, DeepAR yields 97.6% and ARIMA 94.4%.

This improvement arises from the model’s ability to adapt uncertainty estimates across different trajectories and time steps. While deterministic training, based on (11), implicitly assumes a constant error magnitude across all instances, the probabilistic training approach, which optimizes (10), relaxes this assumption by allowing the uncertainty distribution to dynamically adjust across different trajectories and time steps.

The predicted variance in our model captures the total predictive uncertainty learned from the data, without explicitly disentangle epistemic uncertainty (e.g. due to limited data or model uncertainty) from aleatoric variability arising from unobserved external factors. Although such a distinction could be valuable in certain contexts, as done in Bayesian approaches [4, 42], it would require an explicit model of the noise distribution [55]. In the current framework, both the predictive and epistemic uncertainty are treated jointly.

In parallel, we also experimented with sing S-Mamba for $\sigma^{\phi_\sigma}_{1:T}(x_{1:P})$, which led to overestimated uncertainty, indicating that the dynamics of uncertainty are not captured by an SSM. These results are presented in appendix A.

### 3.4. Traffic occupancy (real-world)

As another real-world benchmark, we consider the Traffic dataset [56], which consists of hourly measurements collected by the California department of transportation. This dataset records road occupancy rates from multiple sensors deployed along San Francisco Bay Area freeways and has been widely used in TSF research [14, 38].

Similar to electricity consumption, traffic occupancy reflects human-driven dynamics but is subject to even greater external variability. While periodic patterns emerge from daily commuting routines, unpredictable disruptions such as city events, accidents, and weather conditions play an even more significant role in shaping traffic flow.

**Figure 6.**
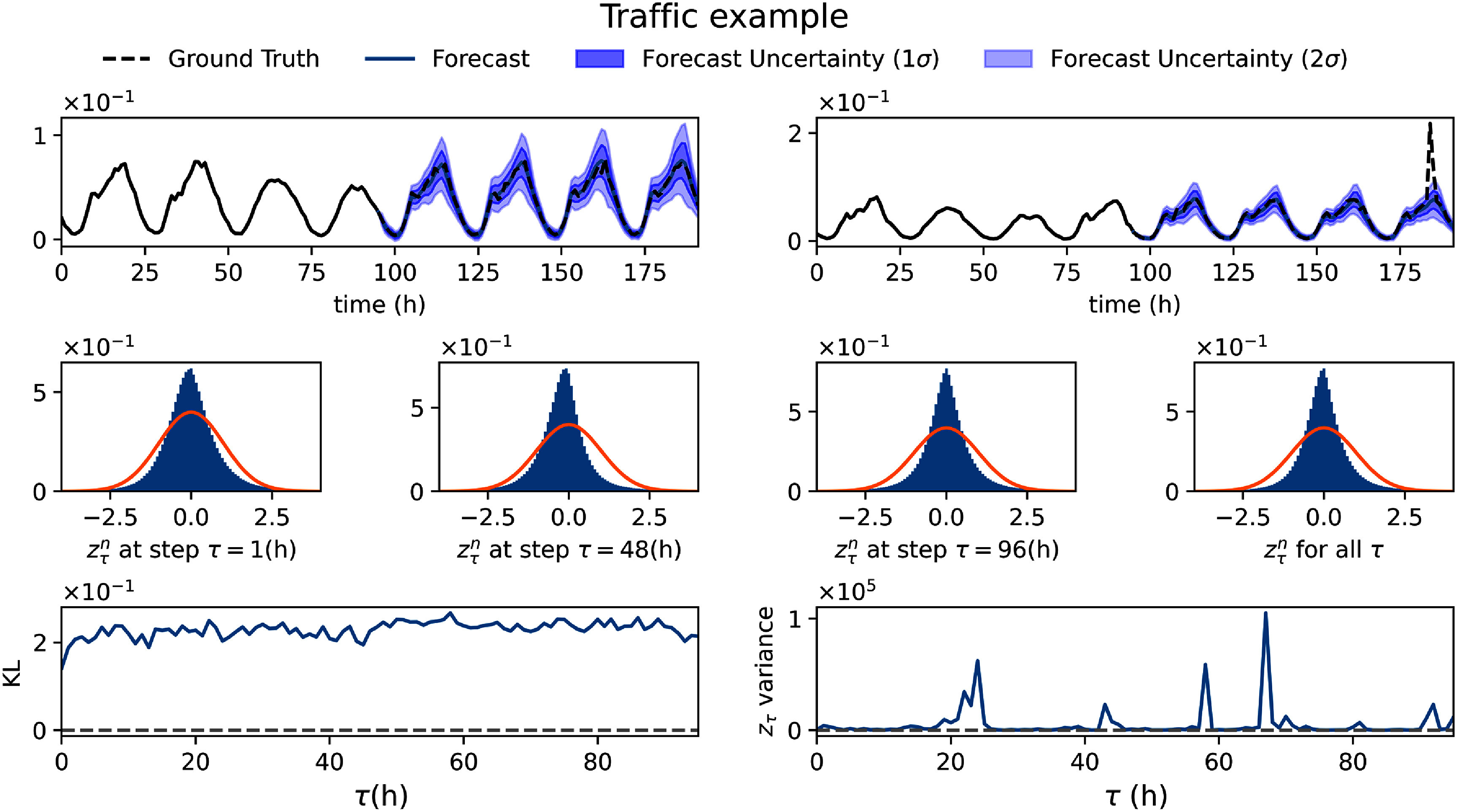
Mamba-ProbTSF in real-world traffic data. The dual-network strategy ensures stable uncertainty estimates using a fully connected neural network for $\sigma^{\phi_\sigma}_\tau(x_{1:P})$. The KL divergence between standardized residuals, $z_\tau^n$, and a standard normal distribution remains on the order of 10^−1^. However, some rare congestion events cause high variance, as seen in the trajectory example (top-right), where a sudden traffic spike occurs. Despite these anomalies, the model maintains reasonable and conservative uncertainty estimates.

In figure 6, we present results from the dual-network strategy applied to the Traffic dataset. The KL divergence between standardized residuals, $z_\tau^n$, and a standard normal distribution remains on the order of 10^−1^. However, the variance of $z_\tau^n$ can become significantly large, which can be attributed to rare but extreme congestion events. This is evident in the trajectory example (top-right panel of figure 6), where a sudden traffic spike occurs due to such an event.

**Figure 7.**
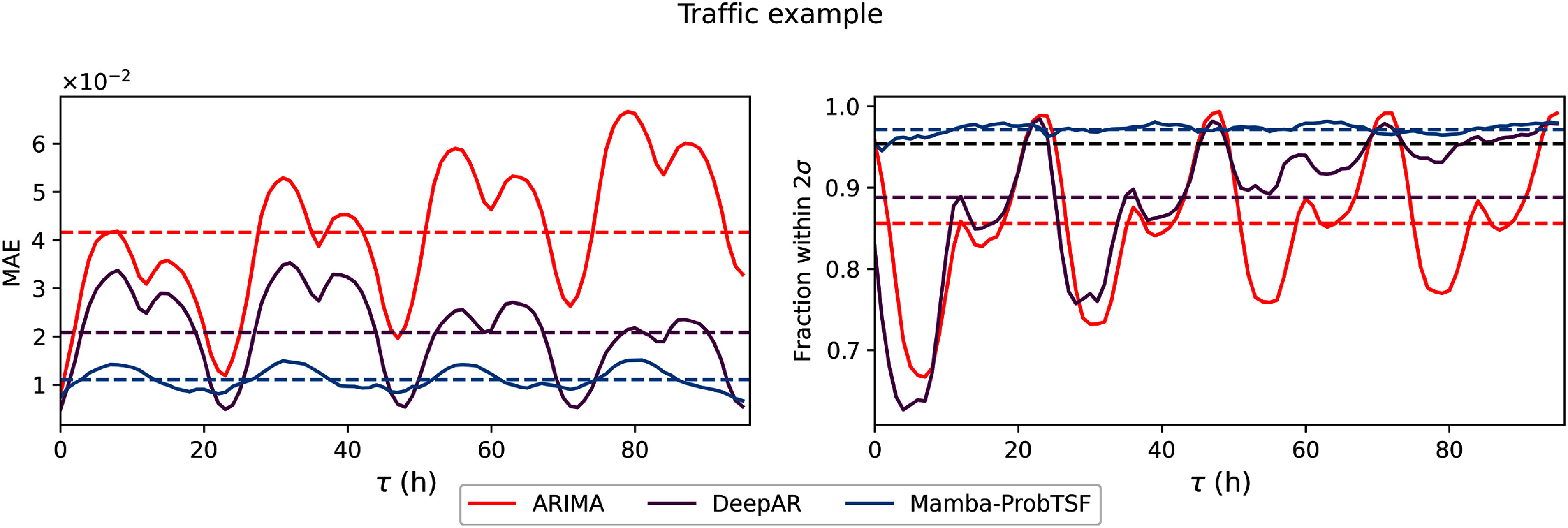
Mamba-ProbTSF provides reliable traffic forecasts with well-calibrated uncertainty. (Left) Mamba-ProbTSF achieves the lowest mean absolute error (MAE) across the forecast horizon, indicating strong point forecasting performance. (Right) We report the fraction of true trajectories falling within the 2*σ* uncertainty interval for each method. Mamba-ProbTSF remains consistently closer to the ideal 95.4% coverage throughout the forecast window, indicating accurate and stable uncertainty estimates. In contrast, all other methods considerably overestimate uncertainty, with coverage well above the ideal and a clear seasonal pattern that mirrors the traffic cycles. This suggests they fail to adapt their predictive intervals to varying traffic conditions, unlike Mamba-ProbTSF, which achieves both accuracy and reliability.

Despite the presence of these outliers, the model maintains conservative uncertainty estimates. In figure 7, we find that the real trajectories fall within the 2*σ* uncertainty interval of Mamba-ProbTSF 97.3% of the time on average, slightly exceeding the expected (95.4%) thus indicating a mild overestimation of uncertainty. However, this remains closer to the target than DeepAR (88.8%) and ARIMA (85.6%), both of which underestimate the range of likely outcomes by a comparable margin. A closer inspection of figure 7 reveals that the fraction of trajectories falling within the predicted intervals for DeepAR and ARIMA fluctuates with a periodic pattern: they tend to underestimate during low-traffic periods and overestimate during high-traffic spikes. This mismatch suggests that Mamba-ProbTSF offers more consistent performance over time, providing reliable and interpretable uncertainty estimates across varying traffic conditions.

Interestingly, in the top-right panel of figure 6, we observe a case where periodicity is present, but the traffic occupancy increases sharply during our forecast. The model successfully captures this behavior, correctly predicting the eventual rise in traffic levels. Notably, Mamba-ProbTSF avoids overconfident predictions during this sharp transition, suggesting that it can handle such rare events without overfitting, while still maintaining accurate and calibrated forecasts during more stable periods.

### 3.5. Performance comparison

To evaluate the performance of Mamba-ProbTSF in both accuracy and uncertainty quantification, we compare it against standard implementations of ARIMA and DeepAR from the GluonTS library. All models are tested on the same prediction tasks and evaluated on the same test sets to ensure consistency.

DeepAR was trained using the same datasets, batch sizes, and maximum number of epochs as Mamba-ProbTSF, providing a direct basis for comparison. However, the two methods differ in how they generate uncertainty: DeepAR samples from a learned distribution at inference time, while Mamba-ProbTSF outputs uncertainty directly through its network as $\sigma^{\phi_\sigma}_{1:T}(x_{1:P})$. This enables Mamba-ProbTSF to provide accurate uncertainty with lower computational cost during evaluation.

ARIMA, in contrast, fits a separate model to each individual time series rather than learning jointly from the full dataset. This per-series estimation hinders large-scale training, as it is difficult to parallelize and does not leverage shared structure across series. Consequently, to include ARIMA in our benchmarking, we constrained all models to a smaller test set containing 4992 trajectories. Nonetheless, ARIMA forecasts are evaluated on the same subset to ensure a fair and consistent comparison with other methods.

**Figure 8.**
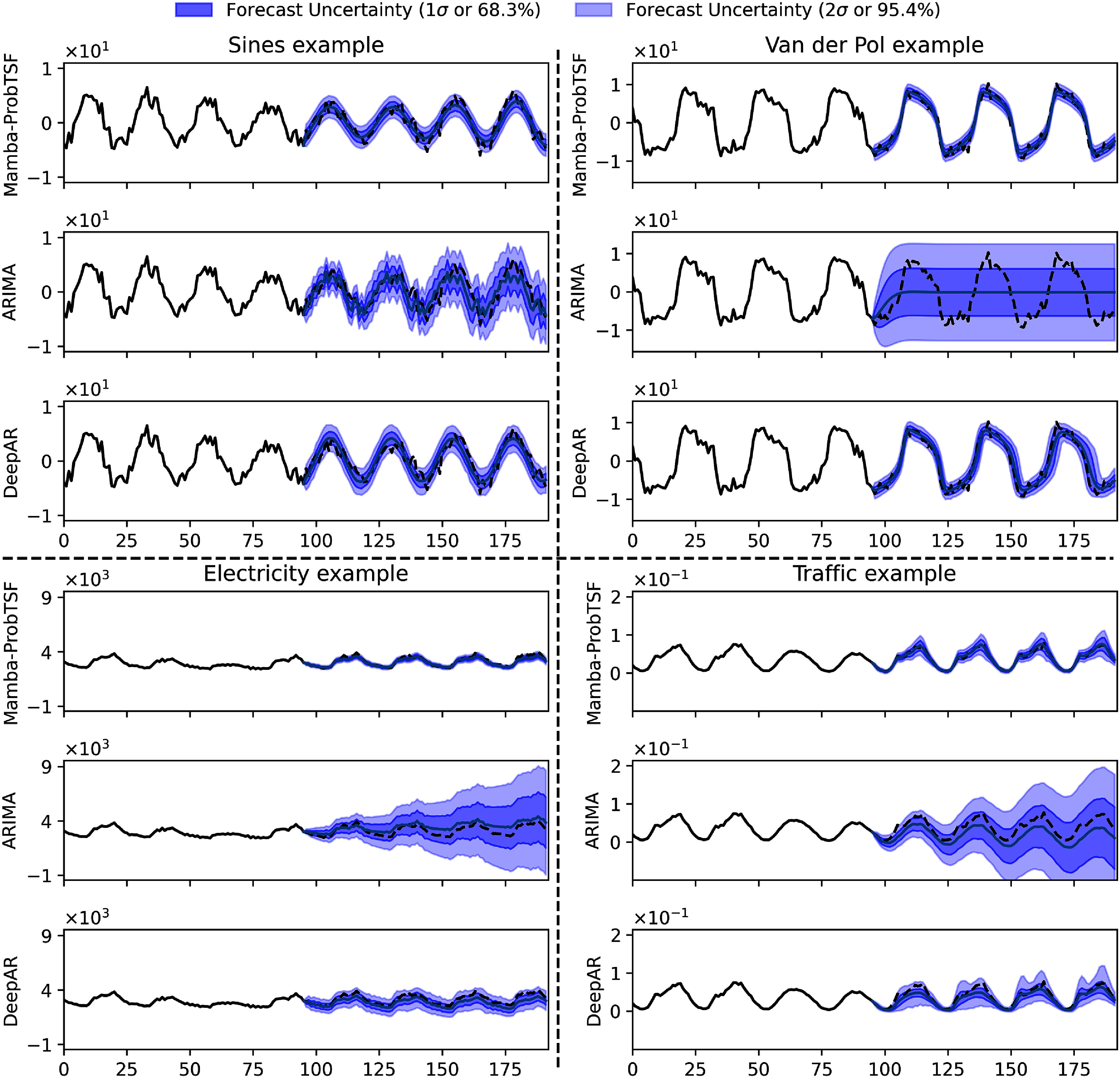
Representative forecast trajectories across different datasets. Each panel shows one forecast trajectory from ARIMA, DeepAR, and Mamba-ProbTSF on the same time series, highlighting both predicted means and uncertainty intervals. Prediction intervals correspond to 1*σ* and 2*σ* (i.e. 68.3%, 95.4%) of the predictive distribution: for ARIMA and DeepAR these are computed from quantiles, while for Mamba-ProbTSF they are computed from $\sigma^{\phi_\sigma}_{1:T}(x_{1:P})$. We provide detail comments on these results in section 3.5. Additional examples are shown in C.

Table 2 summarizes the results in terms of mean absolute error (MAE) and the fraction of trajectories falling within the predicted 68.3%, 95.4%, and 99.7% intervals, corresponding to 1*σ*, 2*σ*, and 3*σ* under a Gaussian assumption. For ARIMA and DeepAR, these intervals are constructed from central quantiles of the forecast distribution (e.g. the 95.4% interval spans from the 2.3rd to the 97.7th percentiles). In contrast, Mamba-ProbTSF defines its intervals using the predicted standard deviation $\sigma^{\phi_\sigma}_{1:T}(x_{1:P})$. Mamba-ProbTSF achieves either superior or highly competitive performance across all datasets and metrics, demonstrating both accurate point forecasts and reliable uncertainty quantification. Figure 8 shows a representative forecast trajectory from each model. On the Sines dataset, all methods perform well. In contrast, ARIMA performs poorly on the Van der Pol system, likely due to its inability to capture nonlinear dynamics. For the Electricity dataset, both ARIMA and DeepAR tend to produce overly wide uncertainty intervals, while in the Traffic dataset this effect is more subtle due to the inherently high variability. Nonetheless, the tendency to overestimate remains evident upon closer inspection and aligns with the quantitative comparisons.

**Table 2.** Mamba-ProbTSF provides forecasts with accurate uncertainty quantification. We compare the mean absolute error (MAE) and the fraction of ground-truth values that fall within prediction intervals corresponding to 1*σ*, 2*σ*, and 3*σ*. These intervals are expected to contain approximately 68.3%, 95.4%, and 99.7% of the predictive distribution, respectively. For ARIMA and DeepAR, the intervals are constructed using central quantiles of the forecast distribution (e.g. the 68.3% interval spans from the 15.85th to the 84.15th percentiles). For Mamba-ProbTSF, the intervals are derived directly from the predicted standard deviations $\sigma^{\phi_\sigma}_{1:T}(x_{1:P})$. Mamba-ProbTSF achieves either the best or highly competitive performance in both point prediction accuracy and uncertainty quantification.

Dataset	Method	MAE	1*σ*	2*σ*	3*σ*
Sines	Mamba-ProbTSF	**0.838**	**0.667**	0.947	0.995
	ARIMA	1.721	0.719	0.951	0.995
	DeepAR	0.986	0.657	**0.953**	**0.998**

Van der Pol	Mamba-ProbTSF	**0.817**	**0.685**	**0.955**	**0.997**
	ARIMA	4.340	0.585	0.961	0.999
	DeepAR	1.128	0.707	0.971	0.999

Electricity	Mamba-ProbTSF	**141.836**	0.794	**0.958**	0.988
	ARIMA	287.398	**0.784**	0.944	0.988
	DeepAR	240.371	0.826	0.976	**0.997**

Traffic	Mamba-ProbTSF	**0.011**	0.884	**0.973**	**0.987**
	ARIMA	0.042	**0.645**	0.856	0.947
	DeepAR	0.021	0.566	0.888	0.986

These qualitative findings are consistent with the empirical fractions of future values falling within the predicted intervals shown in table 2, and further illustrated in figures 5 and 7. To support these comparisons, we also include additional forecast trajectory examples for all datasets and methods in appendix C.

### 3.6. Failure mode: Brownian motion (synthetic)

In this synthetic example, we consider the Brownian motion which presents a failure mode for SSMs. Brownian motion is a stochastic process where randomness is an inherent part of the system’s evolution rather than an external source of noise added on top of deterministic dynamics. Each trajectory in the dataset follows the recursive update:

\begin{equation*} x_t^n = x_{t-1}^n + \xi_t^n,\end{equation*}

where each initial condition, $x_0^n$, is sampled from a uniform distribution on $[0,1]$, and each $\xi_t^n$ is sampled independently from a standard normal distribution for all *t*.

Unlike the previous synthetic datasets, where the underlying system was deterministic with stochastic perturbations, Brownian motion is entirely driven by stochasticity. This introduces a key theoretical feature: given a past trajectory $x_{1:P}$, the conditional probability density function of future values follows

\begin{equation*} p\left(x_{P+\tau} | x_{1:P}\right) = \frac{1}{\sqrt{2\pi \tau}} \exp\left[ -\frac{1}{2} \frac{\left(x_{P+\tau} - x_P\right)^2}{\tau} \right].\end{equation*}

This result implies that the expected value remains at the last observed point, *x_P_*, while the variance grows proportionally to *τ*. This explicitly fits the Gaussian assumption as when we compare with (8), we identify $\mu^{\phi_\mu}_\tau(x_{1:P}) \approx x_P$ for all *τ* and $\sigma^{\phi_\sigma}_\tau(x_{1:P}) \approx \sqrt{\tau}$.

The results obtained from this dataset are shown in the B. There, we evaluate the model’s ability to capture the expected distribution using both S-Mamba and a fully connected linear network for $\sigma^{\phi_\sigma}_{1:T}$. In both cases, we find that the model struggles to fully recover the analytical distribution, with deviations particularly pronounced at both short and long forecasting horizons.

The explanation for this behavior is that SSMs such as Mamba are designed primarily for capturing deterministic latent structures and may not be well-suited for representing stochasticity as an intrinsic part of the dynamic. This highlights the challenge of learning purely stochastic processes with state-space models, the dynamical assumptions described in section 2.2 are insufficient to fully account for the accumulation of uncertainty over time in processes such as Brownian motion. In other words, a dynamics like the one presented in (18), will not be represented as differential equation and linear projection alone as expected by SSMs.

## 4. Discussion

Here, we propose a dual-network architecture for TSF generating both the forecast and its associated uncertainty, brought together in Mamba-ProbTSF. The model is trained using a loss function based on the negative log-likelihood of a Gaussian distribution (10) where one network predicts the mean and the other estimates the square root of the variance. This formulation provides a forecast uncertainty that aligns with a Gaussian-like distribution. The complete code for Mamba-ProbTSF training and the comparisons presented here are available in our GitHub repository [27].

We evaluate the performance of this on two synthetic datasets characterized by known dynamics where the observations are given with additive Gaussian noise, as presented in figures 2 and 3. In those cases, we can observe a near perfect (measured by KL divergence and residue variance) match with the underlying distribution. In real-world examples (figures 4 and 6) we also obtain a good match (KL divergence on the order of 10^−1^) when using S-Mamba for the forecast network and a fully connected network for the uncertainty. While the match was considerably worse than in the synthetic cases, this can be attributed to the fact that real-world dataset variations are not strictly Gaussian.

We nevertheless observe in figure 8 that Mamba-ProbTSF performs robustly across both synthetic and real-world datasets. This is supported by the quantitative results in table 2, where Mamba-ProbTSF yields a fraction of trajectories within the 2*σ* interval that is closer to the target 95.4%. A more complete view can be drawn from the Electricity benchmark in figure 5 and the Traffic benchmark in figure 7, where we observe that Mamba-ProbTSF not only better aligns with the theoretical confidence level, but also maintains consistent behavior over time. Unlike ARIMA and DeepAR, Mamba-ProbTSF does not exhibit seasonal patterns in its uncertainty estimation error.

On the other hand, when applied to processes that accumulate stochastic variation-such as Brownian motion-the Mamba-based model falls short, as discussed in appendix B. This suggests that while the proposed architecture effectively models uncertainty in systems with structured dynamics, additional considerations may be needed to extend its applicability to processes where latent deterministic dynamics do not underlie the process itself. When dynamics are dominated by the accumulation of stochastic noise, it cannot be written as the solution of a hidden differential equation, a fundamental assumptions of SSMs discussed in section 2.2. Addressing this challenge would require rethinking SSM formulations to better accommodate Brownian-like behavior, where uncertainty evolves as a function of stochastic accumulation rather than latent deterministic dynamics. Moreover, this enables the study of mixed cases in which the system follows deterministic dynamics while being influenced by Brownian-like noise [41, 57, 58], as seen in many approaches to molecular dynamics [59, 60].

Yet, given the strong results on structured datasets and real-world data, our study demonstrates the potential of the dual-network approach for probabilistic TSF. Future work could explore hybrid architectures, where the SSM is complemented by another modeling approach that better captures stochastic accumulation. In that sense NN approaches designed to approximate probability distributions, such as normalizing flows [61–63] and flow-based diffusion models [64, 65], could further enhance the model’s robustness in highly stochastic regimes.

## Figures and Tables

**Figure A1.**
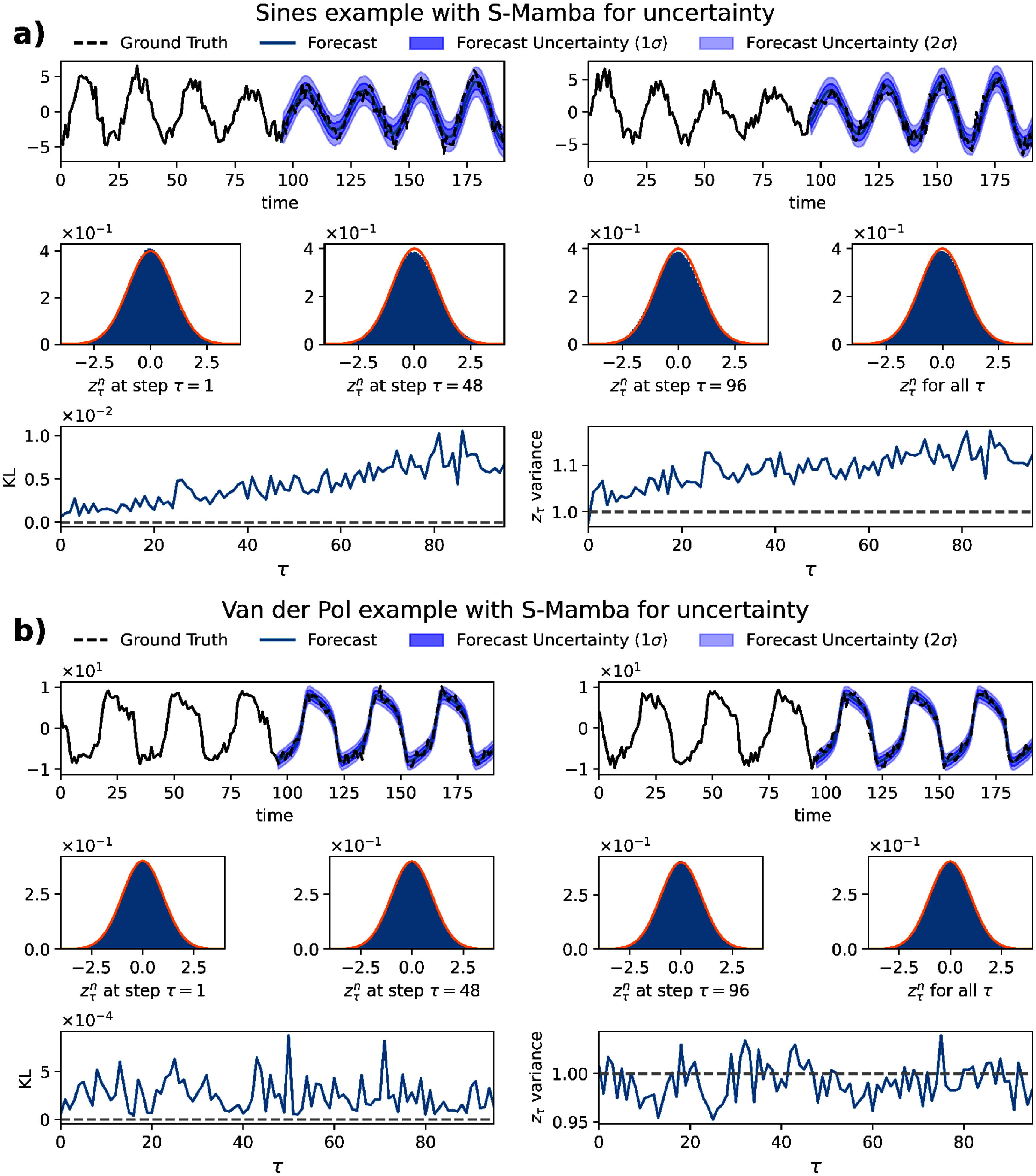
Effect of using S-Mamba with a softplus activation for $\sigma^{\phi_\sigma}_{1:T}(x_{1:P})$ on synthetic datasets. Unlike the main text results, where a fully connected neural network was used for $\sigma^{\phi_\sigma}_{1:T}(x_{1:P})$, here we employ S-Mamba with a softplus activation. Panels (a) and (b) correspond to the sines (figure 2) and Van der Pol (figure 3) examples, respectively. In these cases, the KL divergence and variance of standardized residuals $z_\tau^n$ remain of the same order as in the main text.

**Figure A2.**
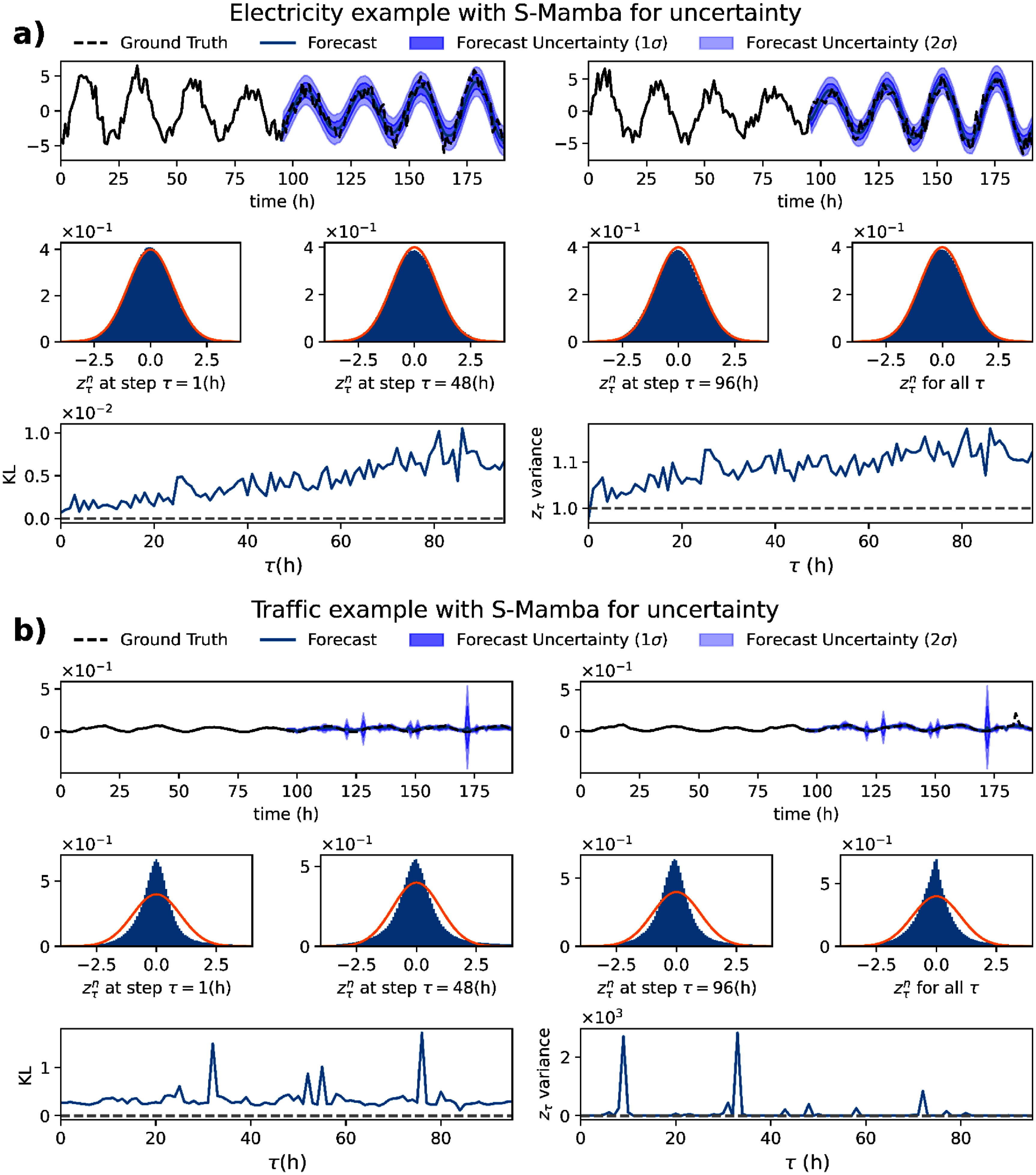
Effect of using S-Mamba with a softplus activation for $\sigma^{\phi_\sigma}_{1:T}(x_{1:P})$ on real-world datasets. Unlike the main text results, where a fully connected neural network was used for $\sigma^{\phi_\sigma}_{1:T}(x_{1:P})$, here we employ S-Mamba with a softplus activation. Panel (a), corresponding to the electricity dataset (cf figure 4), shows dramatic overestimation of the uncertainty, indicating failure of S-Mamba as an architecture for *σ*. A similar overestimation, though less severe, is observed in the traffic dataset (panel (b)).

**Figure B1.**
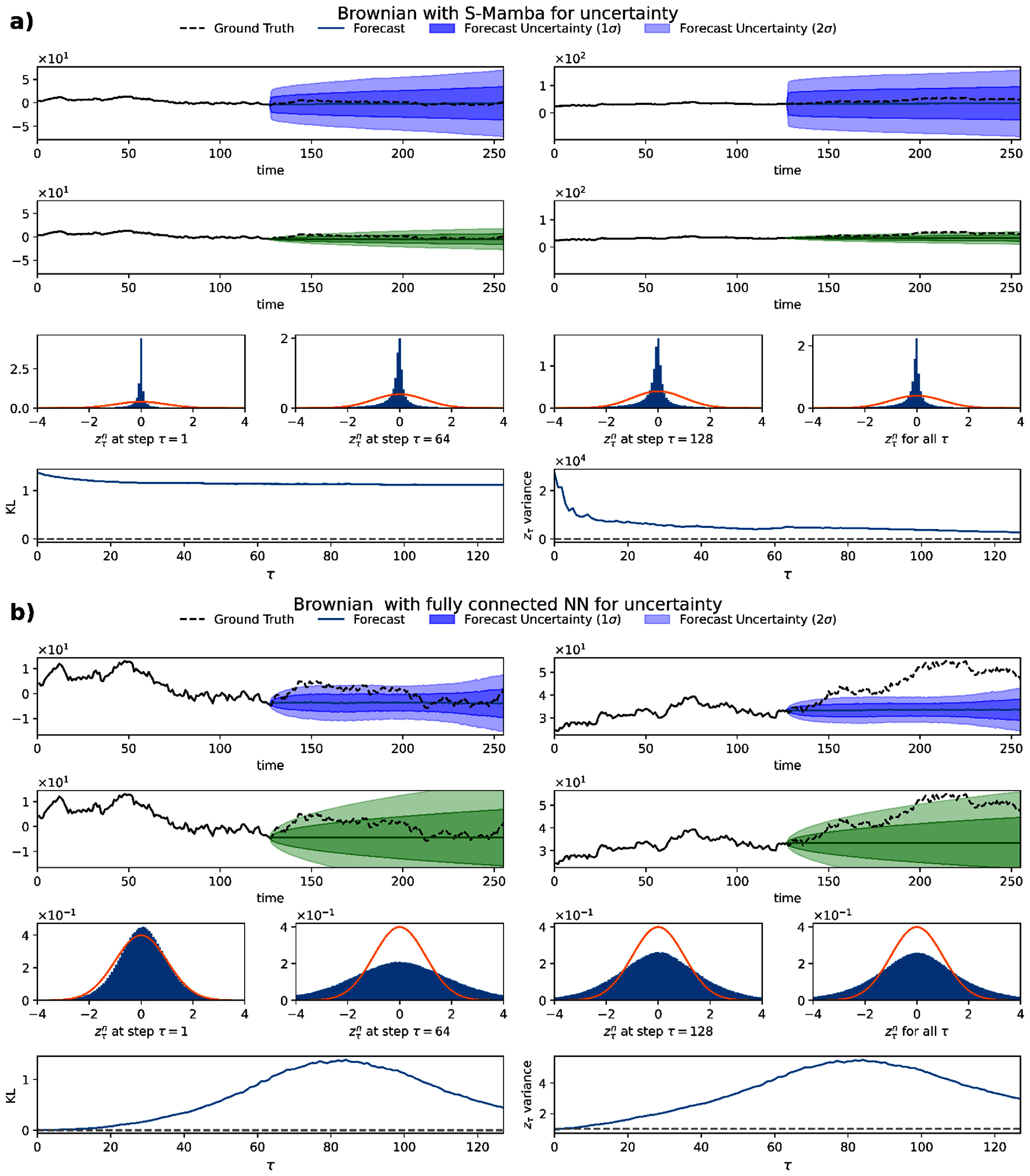
Challenges in modeling Brownian motion uncertainty using neural networks. Brownian motion follows an analytically known probability distribution, where the variance grows as $\sigma^{\phi_\sigma}_{\tau} \sim \sqrt{\tau}$ as expected in (18). The shaded areas represent the predicted distributions (top row) compared to the expected theoretical ones (green shaded on the second row). (a) Using S-Mamba for $\sigma^{\phi_\sigma}_{1:T}(x_{1:P})$ results in significantly overestimated uncertainty, producing forecast intervals that are too broad. (b) A fully connected network for $\sigma^{\phi_\sigma}_{1:T}(x_{1:P})$ yields more reasonable predictions but still fails to precisely recover the theoretically expected distribution. In both cases, the standardized residuals $z_\tau^n$ show a KL divergence close to 1, and the variance of $z_\tau^n$ reaches up to 5, demonstrating the difficulty of capturing purely stochastic processes with state-space models.

**Figure C1.**
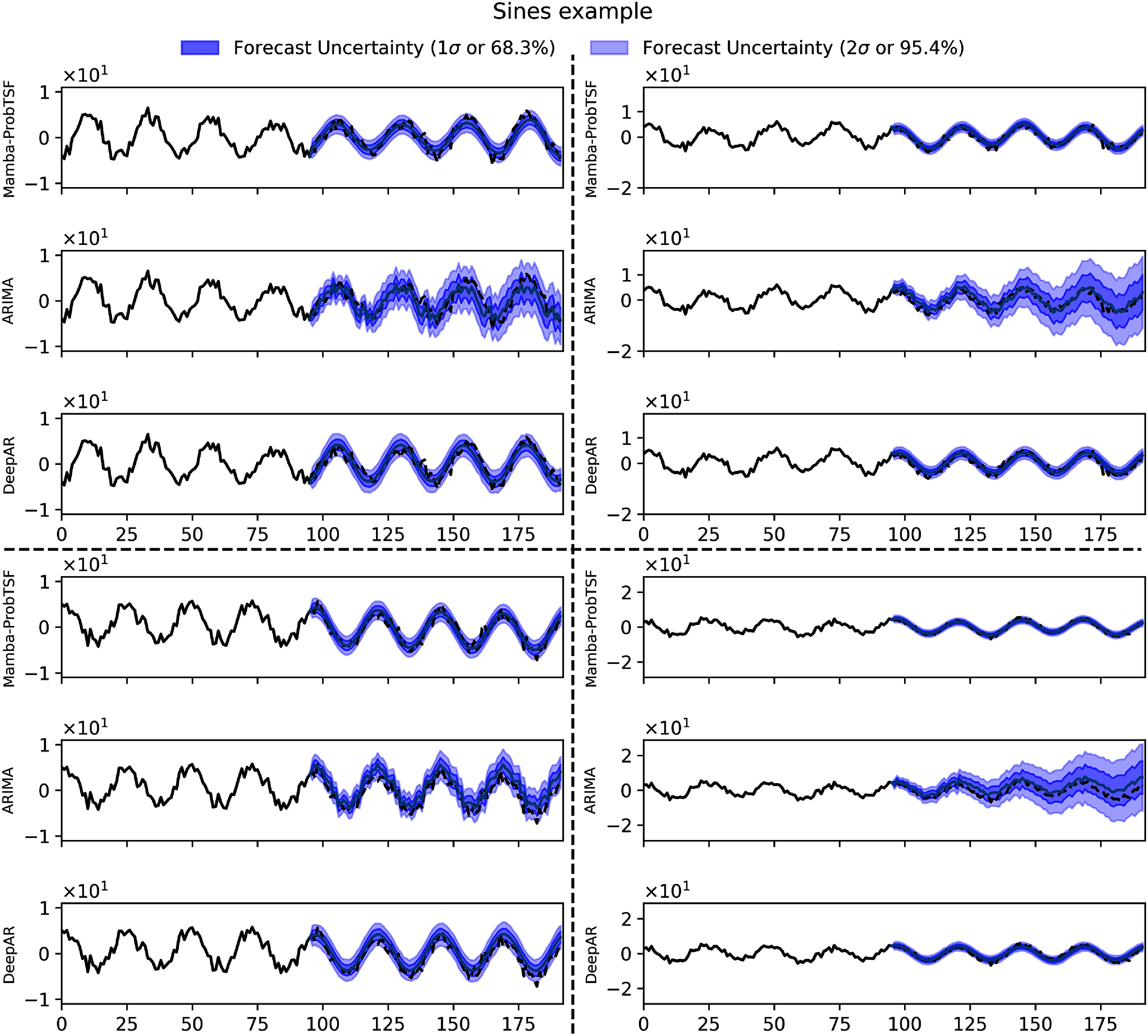
Forecast comparisons—Sines dataset. Multiple trajectories from the periodic Sines dataset. Mamba-ProbTSF maintains consistent predictions and calibrated uncertainty across different input phases, extending the example shown in figure 8.

**Figure C2.**
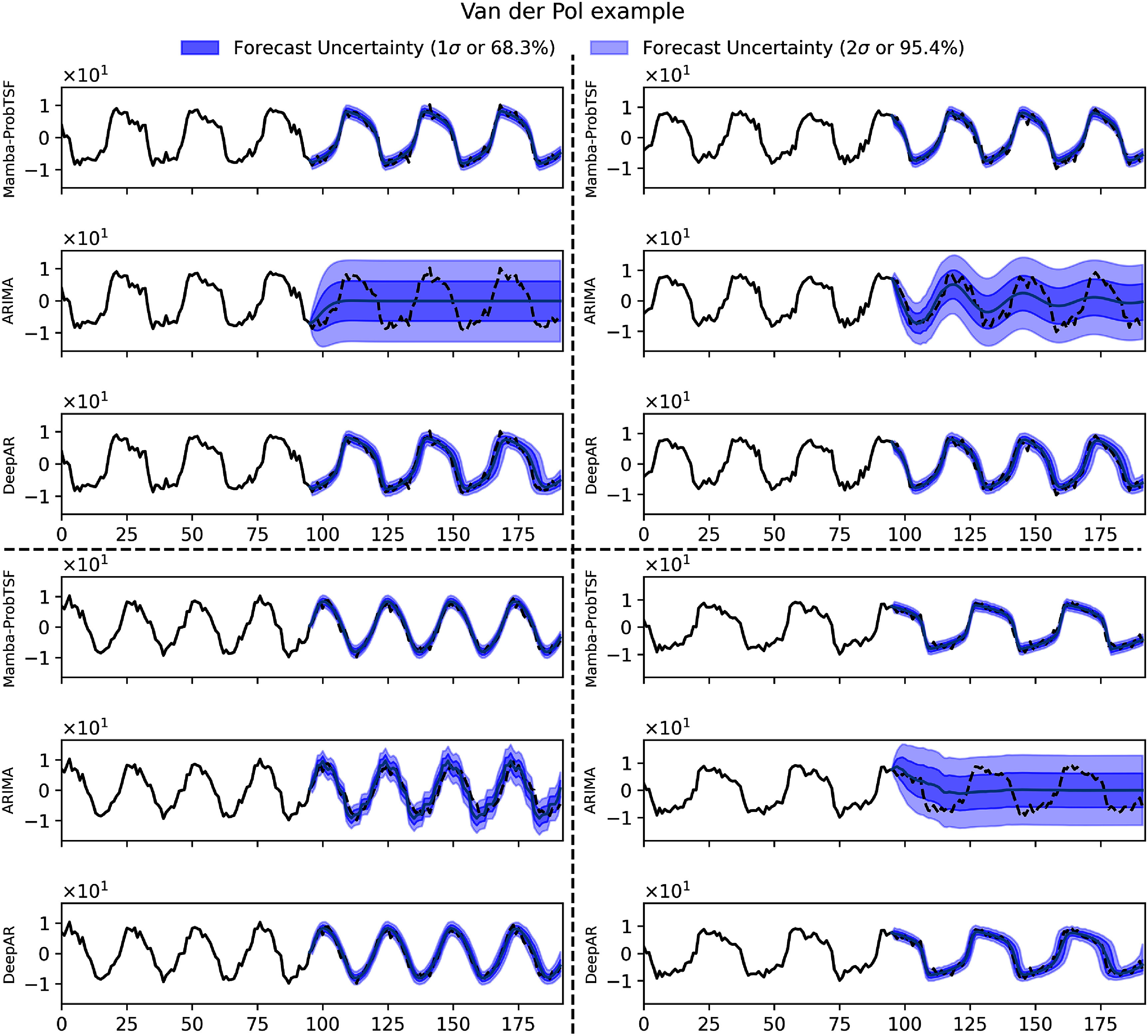
Forecast comparisons—Van der Pol dataset. A set of nonlinear dynamics trajectories from the Van der Pol system. Compared to figure 8, these additional forecasts demonstrate how Mamba-ProbTSF adjusts its uncertainty based on the trajectory regime, particularly near sharp transitions.

**Figure C3.**
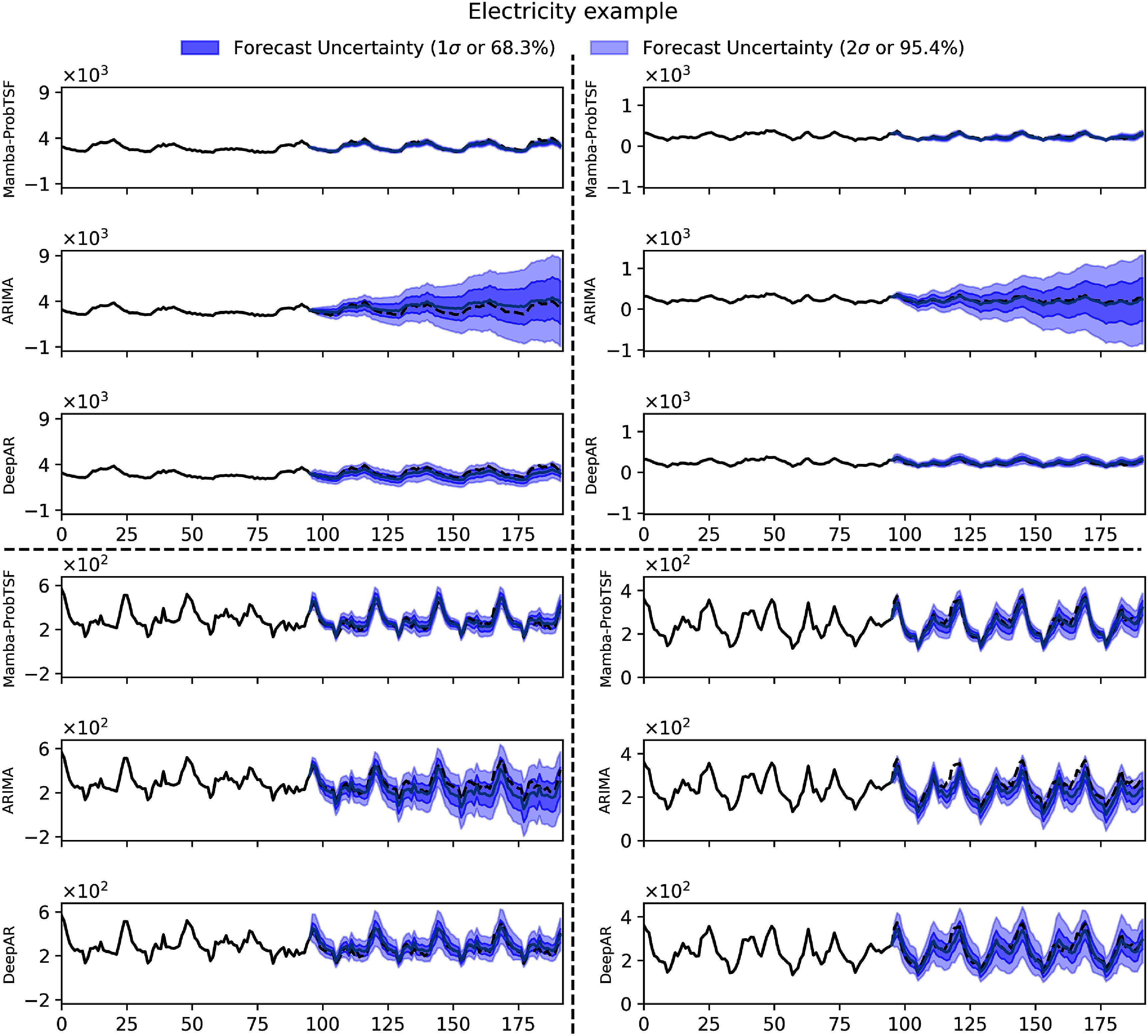
Forecast comparisons—Electricity dataset. Predicted forecasts across distinct electricity consumption series. The model shows robust performance and well-calibrated intervals even across variable load patterns, reinforcing the results shown earlier.

**Figure C4.**
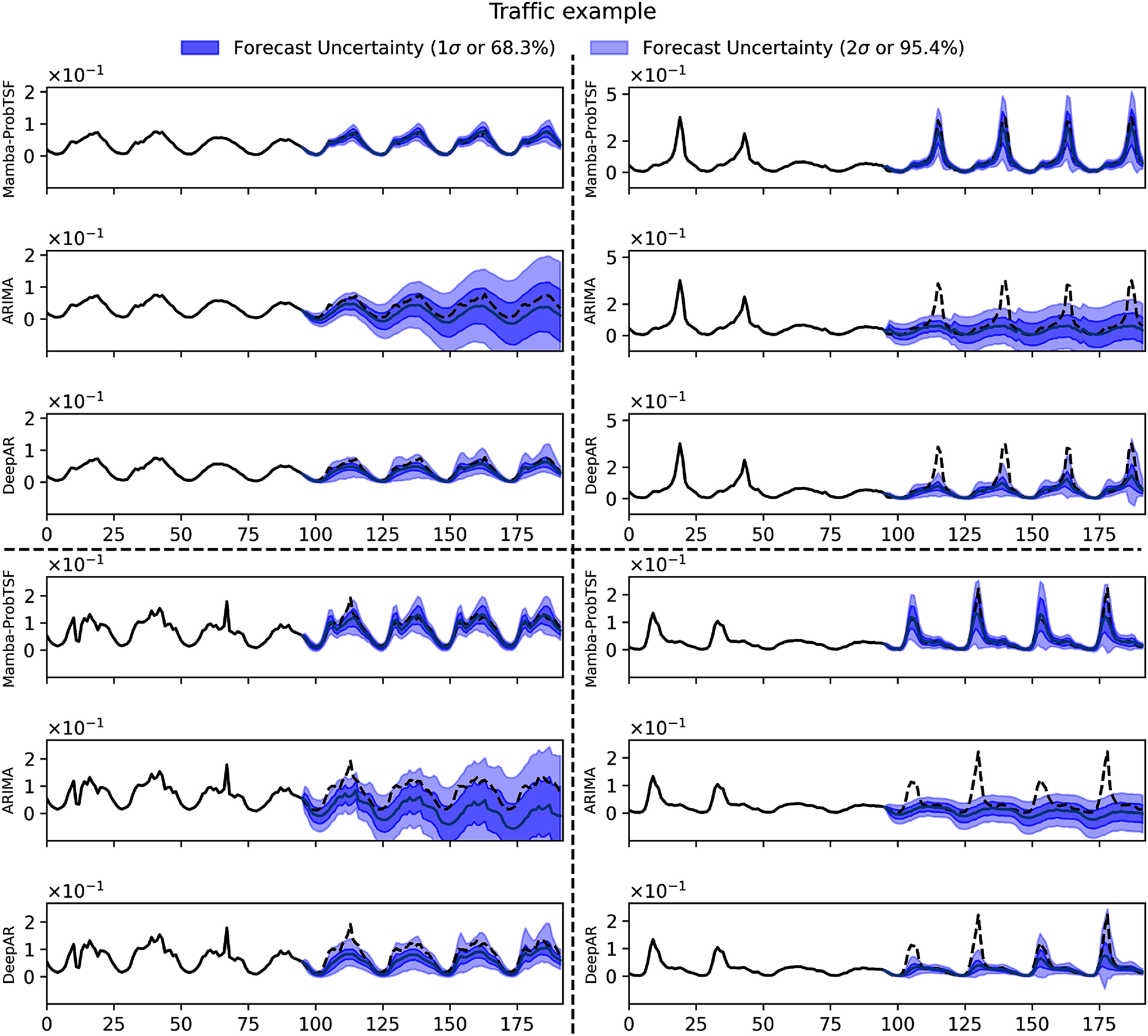
Forecast comparisons—Traffic dataset. Forecasted trajectories under both regular and spiking traffic conditions. These plots extend figure 8, showing that Mamba-ProbTSF remains calibrated and adaptive across a range of test scenarios.

## Data Availability

The data that support the findings of this study are openly available at the following URL/DOI: https://github.com/PessoaP/Mamba-ProbTSF.

## Appendix A Results using S-Mamba for variance

In this supplemental information section, we analyze the results of using the S-Mamba architecture for modeling uncertainty, as proposed in section 2.3 and discussed as a potential source of failure for real-world datasets. We replicate the results from figures 2–4, and 6, but substitute the fully connected neural network previously used for $\sigma^{\phi_\sigma}_{1:T}(x_{1:P})$ with an S-Mamba network.

The updated results are presented in figures A1 and A2. The only architectural change from the main text is the replacement of the fully connected network by S-Mamba for $\sigma^{\phi_\sigma}_{1:T}(x_{1:P})$, while keeping the rest of the training setup identical (as described in section 2.3.2). A softplus activation is still used to ensure positivity of the output.

For the synthetic datasets (figures A1(a) and (b)), corresponding to the sines and Van der Pol examples, the results are comparable to those obtained with the fully connected network. The standardized residuals $z_\tau^n$ approximately follow a standard normal distribution, with KL divergence and variance of the same order as in the main text. Moreover, the estimated variance across different forecast horizons *τ* remains stable, indicating that S-Mamba is capable of capturing meaningful uncertainty in these controlled settings.

In contrast, the results for the real-world datasets (figures A2(a) and (b)) reveal significant shortcomings. In the electricity dataset (panel a), S-Mamba severely overestimates the uncertainty, resulting in excessively wide prediction intervals. This manifests as a variance of $z_\tau^n$ an order of magnitude greater than 1 and a KL divergence two orders of magnitude larger than in the fully connected case (cf figure 4). Similarly, for the traffic dataset (panel b), the model displays an even greater sensitivity to rare deviations, with a KL divergence one order of magnitude larger than that observed in figure 6.

These results suggest that while S-Mamba offers a flexible and powerful architecture for TSF, it may fail to provide reliable uncertainty estimates in real-world settings. In particular, the learned uncertainties deviate substantially from the latent differential equation behavior described in section 2.2.

## Appendix B Results for Brownian motion

As mentioned in section 3.6, the Brownian motion dataset provides a distinct challenge. Unlike the previous synthetic datasets, where the models successfully learned the relationship between uncertainty and time evolution, here we find that neither S-Mamba nor the fully connected network for $\sigma^{\phi_\sigma}_{1:T}(x_{1:P})$ correctly captures the expected variance growth of $\sigma^{\phi_\sigma}_{\tau}(x_{1:P}) \sim \sqrt{\tau}$ — see (18). As shown in figure B1(a), when using S-Mamba for variance estimation, the predicted uncertainty is significantly overestimated, leading to an overly broad forecast distribution. In contrast, figure B1(b) shows that when using a fully connected network, the model produces more reasonable uncertainty estimates but still fails to match the theoretical distribution. This results in standardized residuals $z_\tau^n$ that deviate from the expected standard normal distribution, with KL divergences close to 1 and variance estimates reaching up to 4.

## Appendix C More examples of trajectory forecast

To complement the representative forecasts shown in figure 8, which included one trajectory per dataset, we present additional comparisons below. Now, we have for each dataset a figure displaying multiple forecasted trajectories. Figure C1 refers to the Sines, C2 to the van der Pol, C3 to electricity, and C4 to traffic; demonstrating how model performance and uncertainty quantification vary across different input sequences.

As in the main text, we show the predicted mean (blue), ground truth (black), and shaded uncertainty intervals corresponding to 1*σ*, 2*σ*, and 3*σ* (i.e. 68.3%, 95.4%, and 99.7% coverage), computed from the predicted standard deviation $\sigma^{\phi_\sigma}_{1:T}(x_{1:P})$. on Mamba-ProbTSF and from central quantiles in ARIMA and DeepAR
